# Randomized Trial: D-Glyceric Acid Activates Mitochondrial Metabolism in 50–60-Year-Old Healthy Humans

**DOI:** 10.3389/fragi.2021.752636

**Published:** 2021-10-29

**Authors:** O. Petteri Hirvonen, Heikki Kyröläinen, Maarit Lehti, Heikki Kainulainen

**Affiliations:** Faculty of Sport and Health Sciences, Neuromuscular Research Center, University of Jyväskylä, Jyväskylä, Finland

**Keywords:** mitochondrial activation, re-oxidation, membrane integrity, subclinical inflammation, DGA activation

## Abstract

**Background:** Based on earlier studies, natural metabolite D-glyceric acid (DGA) does not seem to play any role in whole-body metabolism. Nevertheless, one ethanol oxidation-related rat study with controversial results raised our interest. According to preparatory studies for the regulatory approval of DGA, some highly conserved mechanism seems to subtly activate the cellular energy metabolism. Therefore, the present 25-days double-blind human study with placebo control was initiated.

**Purpose:** The main target in the present study with 27 healthy 50–60-year-old human volunteers was to find out whether an “acute” 4-days and a longer 21-days *exogenous* DGA regimen caused moderate activation of the mitochondrial energy metabolism. The simultaneous target was to find out whether a halved dose of DGA continued to be an effective regimen.

**Main Findings:** The results revealed the following statistically significant findings: 1) plasma concentrations of metabolites related to aerobic energy production, especially lactate, were strongly reduced, 2) systemic inflammation was lowered both in 4- and 21-days, 3) mitochondria-related mRNA expressions in circulating immune cells were noticeably modulated at Day4, 4) cellular membrane integrity seemed to be sharply enhanced, and 5) cellular NADH/NAD^+^ -ratio was upregulated.

**Conclusion:** Mitochondrial metabolism was clearly upregulated at the whole-body level in both 4- and 21 days. At the same time, the effect of DGA was very well tolerated. Based on received solid results, the DGA regimen may alleviate acute and chronic energy metabolic challenges in main organs like the liver, CNS, and skeletal muscles. Enhanced membrane integrity combined with lower systemic inflammation and activated metabolic flows by the DGA regimen may be beneficial especially for the aging population.

**GRAPHICAL ABSTRACT:**
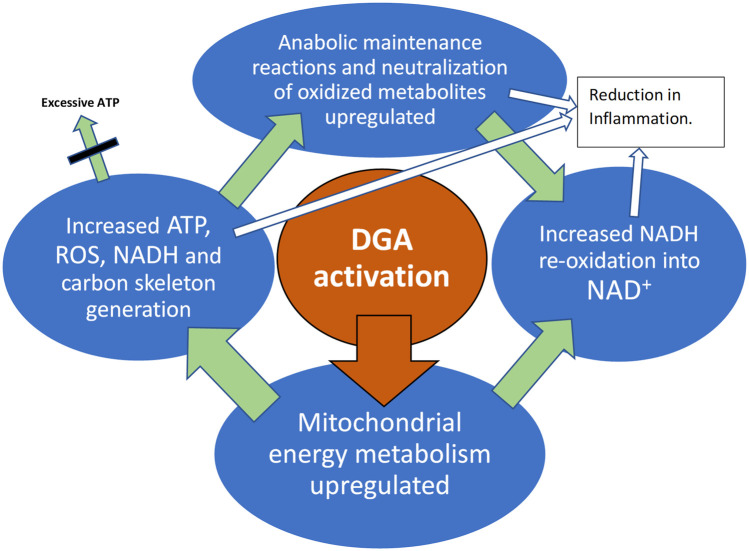
DGA activation materializes in all active tissues like muscles, immune system, and the liver.

## Highlights

Oral D-glyceric acid (DGA) administration causes both fast and lasting positive metabolic effects in healthy 50–60-year-old humans. Overall mitochondrial metabolism was activated by the increase in intracellular DGA concentration. Both the OXPHOS and NADH using anabolic reactions were upregulated in the whole-body and particularly in immune cells and hepatocytes. Plasma lactate was strongly and statistically extremely significantly reduced. Subclinical inflammation measured by 3 independent methods was reduced both in the 4- and 21-days treatments. Cellular membrane integrity seemed to be sharply improved. Creatine kinase and aspartate aminotransferase release to plasma was lowered sharply and statistically very significantly already in 4-days.

## Introduction

D-glyceric acid (DGA) is a natural organic acid present in very small amounts in vertebrates and plants. Nevertheless, there are only a few scientific studies on this small metabolite. Due to its small size and low, varying, concentrations even the measurement of exact DGA concentration from fluids, and tissues at physiological levels is somewhat challenging (Hoffmann et al., 1989). In 27–39 year-old adults, the DGA concentration in plasma was on average only 0.3% of the lactate concentration (Hoffmann et al., 1993). As a small monocarboxylic acid, DGA molecules distribute through monocarboxylate transporters from blood circulation into tissues. Interestingly in pediatric patients, the ratio of DGA concentration in cerebrospinal fluid to plasma seems to be clearly higher than that of organic acids in general (Hoffmann et al., 1993). This may indicate that the diffusion of DGA through the blood-brain barrier as well as other membranes is not totally fluent.

The main enzyme that metabolizes DGA in humans and animals is glycerate kinase (GLYCTK).Glyoxylate reductase hydroxypyruvate reductase (GRHPR) can also oxidize DGA. GLYCTK and GRHPR are widely expressed and active in all tissues (www.proteinatlas.org/). GLYCTK enzymes possess several splice variants that are localized in cytosol and in mitochondria (Guo et al., 2006). According to Uniprot Knowledgebase, 4 splice variants are located in mitochondria and 3 in cytosol. GLYCTK kinase reaction consumes one ATP and simultaneously phosphorylates DGA into glycolytic intermediate 2-phosphoglycerate. There is a rare inborn error called D-glyceric Aciduria that likely relates to some deficiency in GLYCTK (Sass et al., 2010).

Cellular synthesis of DGA occurs mainly from D-glyceraldehyde (D-GALD) via aldehyde dehydrogenase enzymes. These enzymes are often mitochondrially located (Stagos et al., 2010). D-GALD is a product of fructose catabolism, which occurs mainly in the liver and to a lesser extent in the intestines. Interestingly, skeletal muscles and many other organs possess GLUT transporters that are specified into fructose intake from plasma, indicating clearly that D-GALD producing fructose catabolism also occurs to some extent in skeletal muscle (Zierath et al., 1995; Scheepers et al., 2005). In normal physiological conditions, D-GALD is phosphorylated in the cytosol by triose kinase and it enters glycolysis.

In earlier studies, the effects of DGA administration have been linked to ethanol oxidation (Eriksson et al., 2007; Habe et al., 2011). Oxidation of ethanol was shown to be accelerated by a substantial 25% in male rats when acute 500 mg/kg or 100 mg/kg DGA dose was given intraperitoneally 1 hour before the ethanol dose (Eriksson et al., 2007). In the same article (Eriksson et al., 2007), a 3-weeks non-acute DGA administration experiment within the chow was also reported (doses 0, 100, 500, or 1,000 mg/kg of DGA). In this 3-weeks non-acute DGA administration experiment ethanol oxidation was surprisingly not increased. A consistent explanation for these apparently contradicting results was likely a whole-body activation of (mitochondrial) energy metabolism after the acute DGA dose in the studied rats. It increased energy metabolic substrate demand by the peripheral tissues from the liver that caused a mild energy deprivation in the hepatocytes 1 h after the acute DGA dose. Accelerated ethanol oxidation replenished hepatic energy homeostasis (NADH/NAD^+^ -ratio) in the DGA treated rats. Habe et al. (Habe et al., 2011) studied DGA in gastric cells *in vitro* after 2—3% ethanol-dosed medium. In their experiments, cell viability increased in 72 h by certain doses of DGA, LGA, or racemic DL-GA.

The aim of the present study was to find out direct and indirect indications of the activation of mitochondrial metabolism by the use of DGA. When feasible, the tissues affected, and the timing of the response were also evaluated. Simultaneously, we wanted to investigate possible signs of a more permanent mitochondrial activation such as happens after sustained adaptation to a more active physical lifestyle. That kind of pro-energetic effect may lead to positive changes in health risk factors such as deteriorated cell membranes integrity and elevated systemic inflammation (Kim et al., 2019; Tofas et al., 2020; Dias and Nylandsted, 2021).

## Materials and Methods

Altogether 30 healthy participants aged 50–60 years were carefully selected out of 45 healthy and suitable volunteers to form the present study group. The volunteers were recruited through 600 letters sent randomly to age-matched men and women from the area of Central Finland. All the participants were informed of the experimental design, and the benefits and possible risks that could be associated with the study prior to signing an informed consent to voluntarily participate in the study. All studies were conducted in line with the statement of the Ethical committee of the Central Finland Health Care District (Dnro 1U/2019, KSSHP).

### Characteristics of the Study Group

The age group of 50–60 years was chosen because at that age systemic inflammation markers are on average somewhat elevated even in apparently healthy persons (Wyczalkowska-Tomasik et al., 2016). BMI of the participants was restricted to 18.5–32.0 so that there could not be clearly under- or overweight participants. All participants were Caucasians (Table 1).

**TABLE 1:** Characteristics of the study group.

Day0 metrics	Main study group	DGA group	Placebo group
Average Age	56 years (from 50.3 to 60.9)	56.5	55.2
Average BMI	25.3 (from 20.1 to 31.7)	25.0	25.8
Avg. VO_2_max	35.5 (from 21.8 to 48.8)	35.1	36.1
Female/Male	16 females and 11 males	10/7	6/4

Notes: unit in BMI = weight in kg/(length in meters)^2^, unit in VO_2_max = O_2_ ml/kg/min. VO_2_max test was based on indirect measurement with a bicycle ergometer (Santtila et al., 2013). All participants executed this routine test of the JyU Sports Laboratory successfully.

Any history of cardiovascular diseases was an exclusion criterion. Also, those persons who had to travel extensively during the study were excluded. Normal and stable behavior during the study was enhanced by personal diaries and reminder emails. Morning interviews were carried out individually when participants arrived at the study site within a minimum of 30 min before the first blood sample. Also, the health status of the participant, comparable circumstances, and the timing of the last dose were always checked. All the participants who came to the Day 0 measurements completed the whole study. Nevertheless, three of the selected persons canceled at the last minute before the Day 0 measurement due to mild flu or similar symptoms.

### Study Setup and Measurements

**FIGURE 1:**
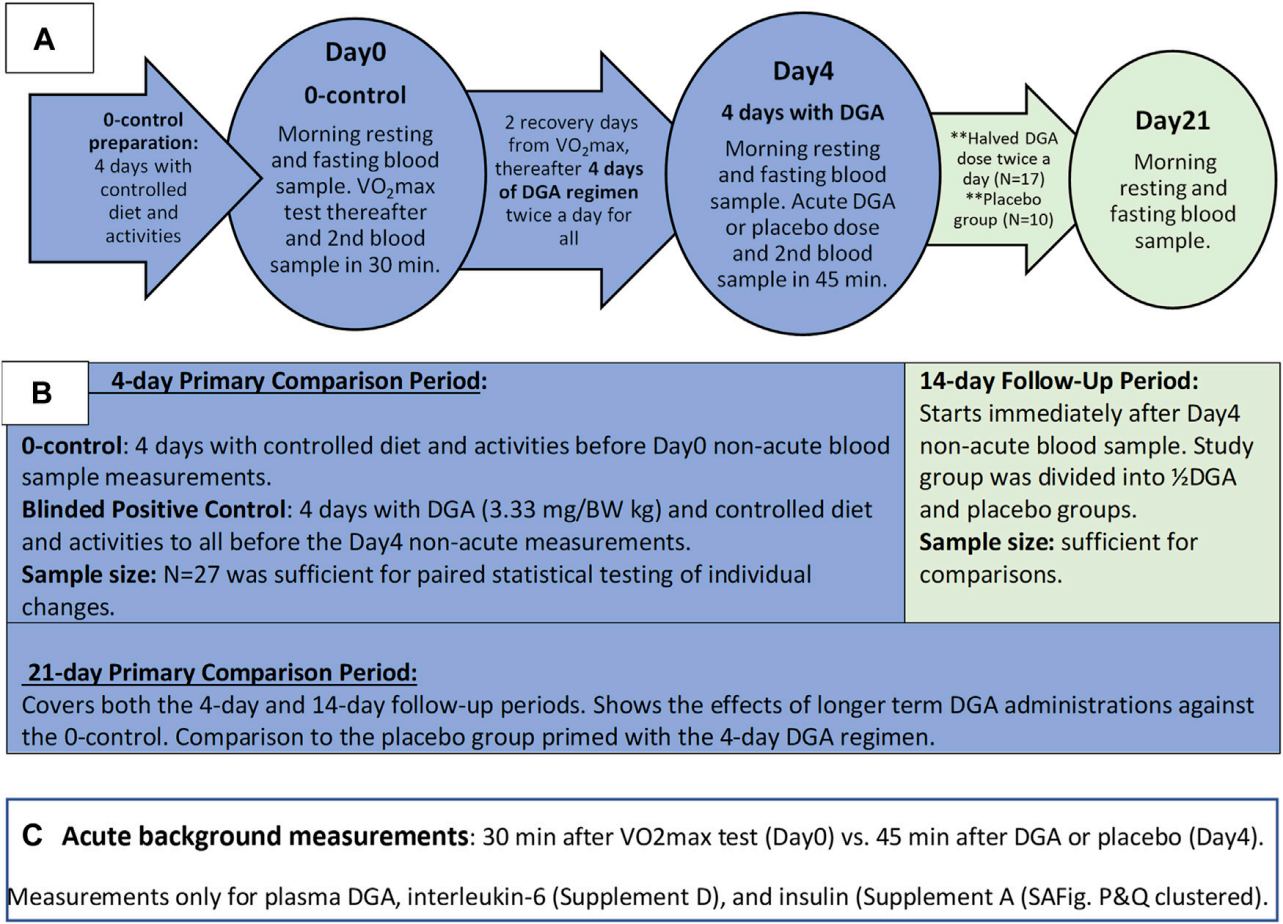
Outline of the Study: Timelines and measurements **(A)**, main phases of study **(B)**, and the acute measurements **(C)**.

The test setup was double-blinded. Measurements were always performed on the same weekday (Friday or Saturday) for each participant. To achieve more comparable same-weekday measurements, we added two recovery days after the Day0 VO_2_max measurements (Figure 1A). For simplicity, we call the second measurement day the “Day4” because it was taken after the 4 days of DGA regimen even though the actual day was the 7th.

The placebo group was chosen randomly among women and men separately beforehand. The existence of the placebo group throughout the study was informed to the participants. In practice, the number of placebo-treated participants was zero until the Day4 measurements. From that day onwards, the number of participants in the placebo group was increased to 10, i.e., the placebo group existed *de facto* only for the 2-weeks follow-up period (Figures 1A,B).

All morning blood samples were taken 12 h after the last DGA or placebo dose, i.e., non-acutely. Blood samples for each participant were always taken at the same time in the morning (+/−2 min). Additionally, throughout the whole study period fully normal but stable living conditions were required.^1^

#### Test Substances and Doses, Preparations, and Administration

D-glyceric acid calcium salt (DGAcs) and placebo (E509/calcium chloride) were dissolved into 1.5 L bottles of water beforehand for each participant. The calculated dose of DGA or placebo was to be drunk in the morning and the evening. In the placebo group, there was an equal molar amount of calcium with water. DGA was produced and purified into calcium salt at a molecular level by VTT Technical Research Centre of Finland (VTT) and Replicon Health Oy in collaboration (Habe et al., 2009). On top of VTT, the purity of the batch was tested by the Finnish Food Authority (residues and concentration), and Pharmatory Oy for enantiomers. Before this study, part of the batch was used in extensive commercial rearing experiments. In the hepatocyte study (Supplementary Presentation 3), used DGAcs were from Sigma-Aldrich (3,67,494).

Selected doses, regimens, and measurement timings in the present study were based on earlier *in vitro* and *in vivo* pre-tests related to the regulatory acceptance processes of DGA. The effective dose of DGA for the first 4-days was 3.33 mg per kg body weight twice per day. That dose was pre-tested in small-scale human piloting as a natural food additive (Hirvonen et al., 2015). In the 14-days follow-up period the DGA regimen was reduced to half because we wanted to explore the smallest sufficient dose for humans. (As a comparison, the highest DGA dose per kg body weight (BW) in the 3-weeks rat experiment (Eriksson et al., 2007) was more than 200-times the dose used in the current human study during the follow-up period. As reported in the article (Eriksson et al., 2007) “no toxicity” was observed in studied rats at any of the doses.)

#### Blood Samples

Blood samples were drawn from the antecubital vein of each participant, always at the same time in the morning. The samples were immediately cooled and centrifuged in heparin plasma tubes and stored in 2 ml portions at −80°C. Plasma samples were analyzed in Nightingale Health Oy (Nuclear Magnetic Resonance (NMR) technology with regulatory approval for diagnostics) (Soininen et al., 2015), except for CK, AST, and ALT that were analyzed in Synlab Finland with clinically accredited standard methods. Insulin, hsCRP, and IL-6 were measured at the JyU laboratory using validated kits, and plasma DGA concentration in VTT using gas chromatography-mass spectrometry technique.

#### RNA Sequencing

mRNA expressions were measured from white blood cells (WBCs). WBCs samples were taken at the same time as morning fasting and resting blood samples. Whole-genome mRNA sequencing was conducted by the Institute for Molecular Medicine Genomics Unit (FIMM/University of Helsinki) (Costello et al., 2018). Collection of whole blood samples into PAXgene Blood RNA Tubes and extraction of mRNA was conducted according to manufacturer’s instructions.

### Human *in vitro* Side Study With Primary Hepatocytes

In our pilot studies with human primary hepatocytes and rat primary cortical neurons, it was noticed that energy consumption seemed to be activated in the DGA treated cells compared to 0-controls (Hirvonen et al., 2015). To find out how energy consumption was increased, we measured NAD+/NADH -ratio from the hepatocytes of three human donors. The results are scientifically reported for the first time in Supplementary Presentation 3.

### Statistical Tests and Sufficient Number of Observations

Each person was used as their own control. That way individual “noise factors” could be eliminated and we were able to pairwise test whether the intraindividual responses were similar among study persons. Furthermore, to achieve statistically unambiguous results all blinded participants were in the same comparison group during the first week. Pre-assessment on the sufficient group size was based on the results from our pilot tests and related volatilities. Among our relatively homogenous study group with fully comparable intraindividual measurement points, the selected group size turned out to be clearly sufficient.

Statistical tests were conducted using IBM SPSS statistics software and Microsoft Excel. Presented statistical test results are mostly from parametric Student’s *t*-tests. When *N* < 15, we checked the normality (Routledge, 2020) of the underlying data using the Kolmogorov-Smirnov test. Non-parametric Mann-Whitney *U*-test or SIGN tests were used when clearly needed. *p*-value <0.05, <0.01, and <0.001 in a one-sided *t*-test were considered statistically significant, very significant and extremely significant respectively. All presented tests were predetermined or derived from predetermined test settings.

## Results and Discussion

**FIGURE 2:**
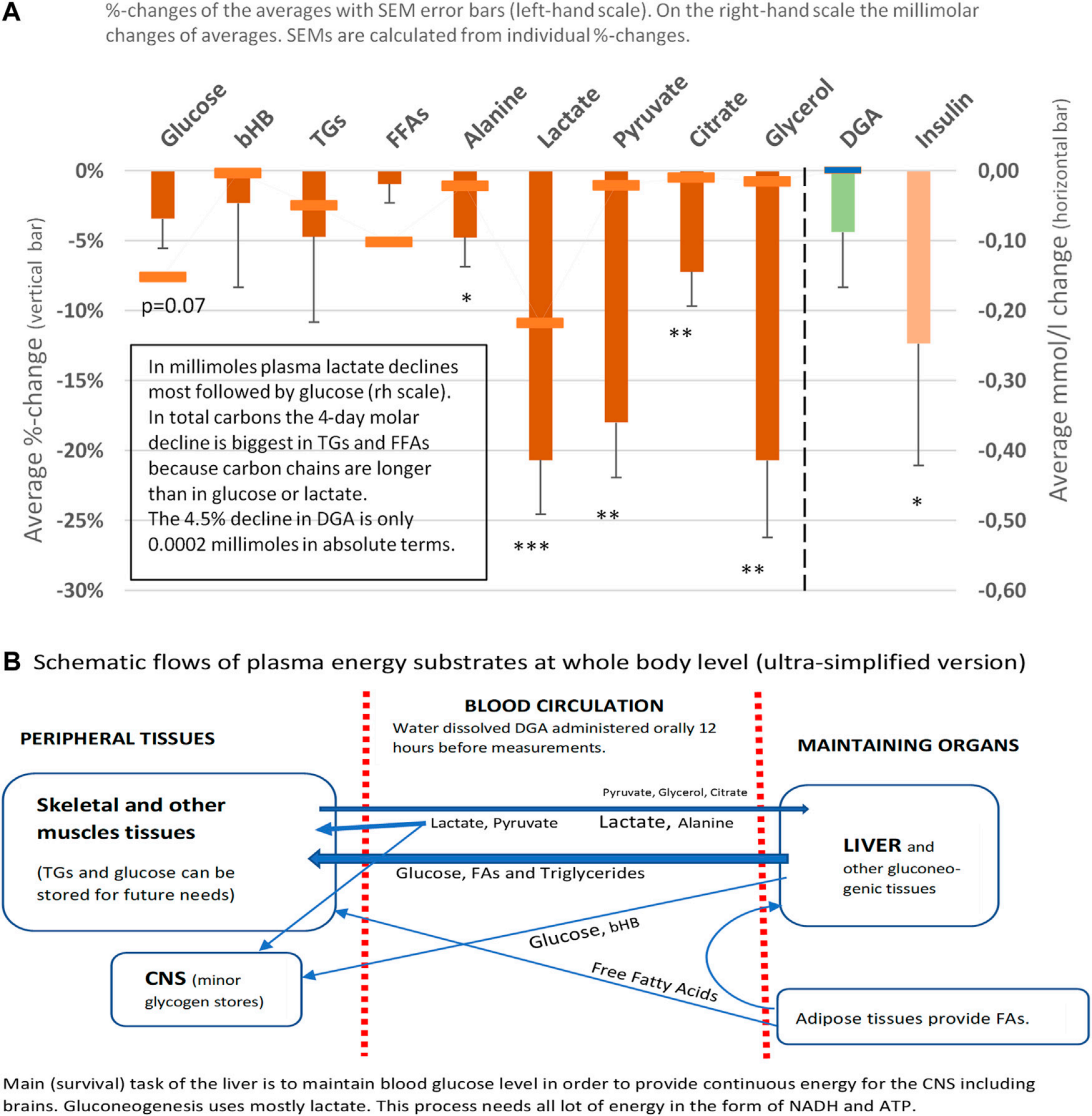
Representative energy substrates under the 4-days DGA regimen, 12 h from last DGA dose, and DGA and insulin **(A)**. Schematic flows of all reported plasma energy substrates at whole-body level **(B)**. Statistical tests are based on intra-individual changes (paired *t*-tests). Error bars are standard errors of the mean (SEM) of the individual changes.

**FIGURE 3:**
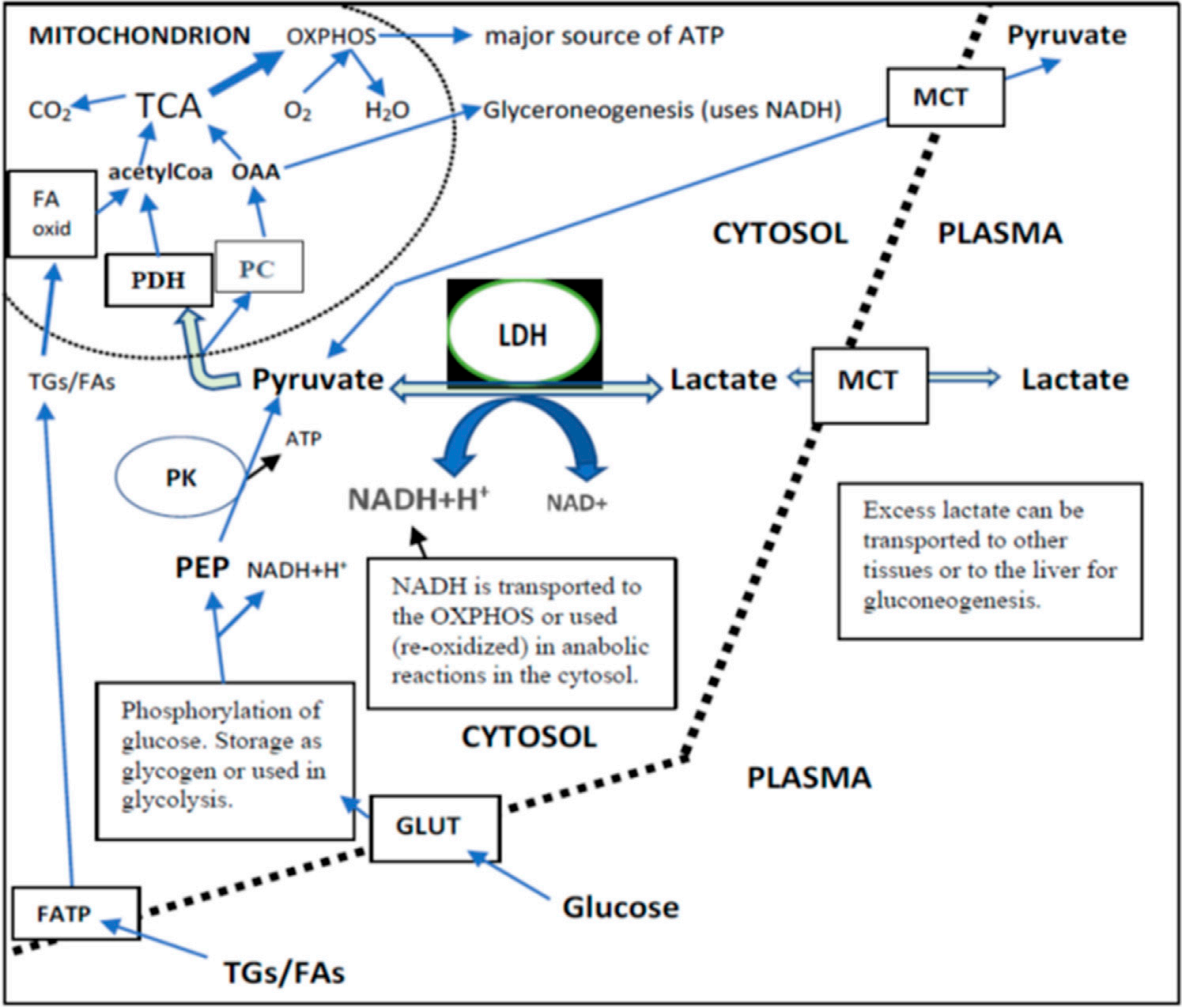
Lactate, pyruvate, glucose, and TGs/FAs inflow from plasma and their intracellular metabolism in a cell that can use both FAs and glucose as the main source of energy. Independently of their final use in metabolism (liver or peripheral tissues), the intracellular direction of lactate via pyruvate is towards mitochondria. This is because the cytosolic PK reaction is irreversible. TCA produces most of the NADH for OXPHOS. Glycolysis produces 2 NADH per one glucose. There existed a very strong correlation between the changes of plasma pyruvate and lactate independently of placebo or DGA treatment (>0.90). This correlation strongly indicates that MCTs are able to balance plasma membrane concentration differences efficiently.

**FIGURE 4:**
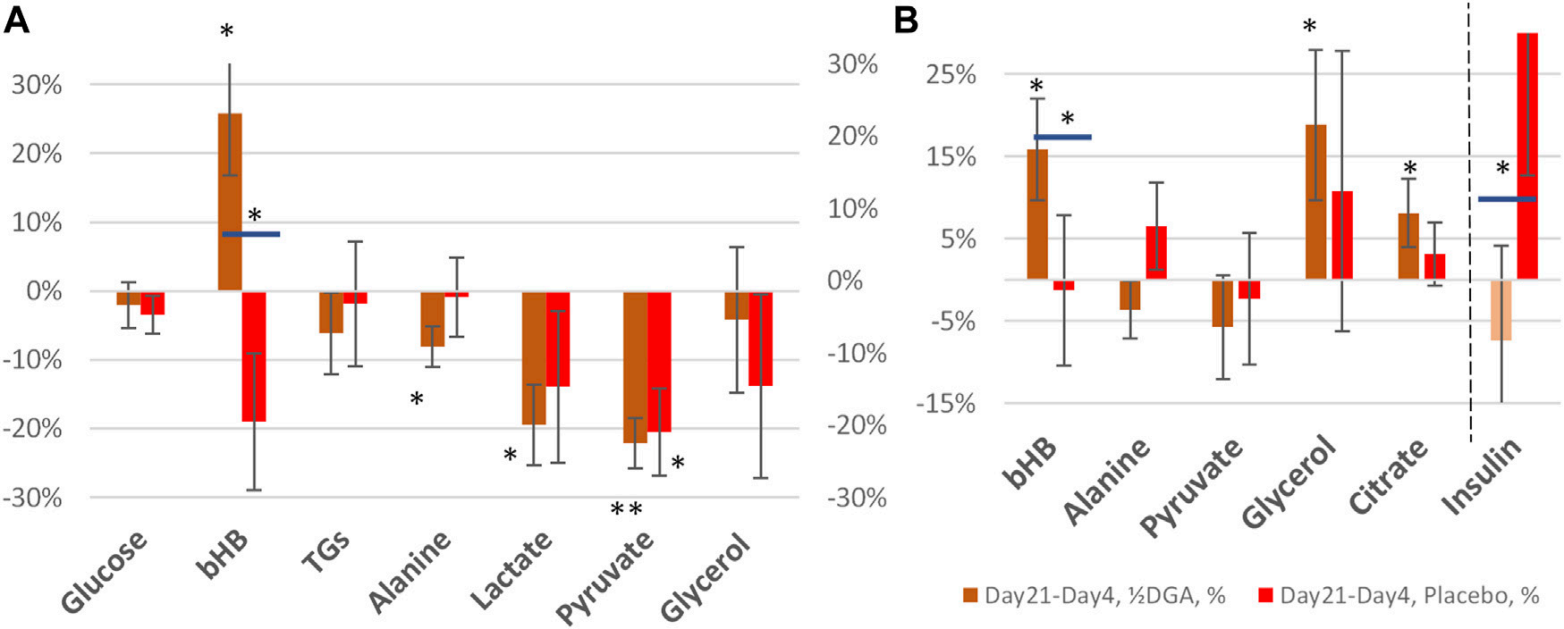
21 days **(A)** and 14 days **(B)** %-changes in selected plasma energy metabolites and insulin. Statistical tests are based on intra-individual changes. In Figure 4A the changes are from Day 0 and in Figure 4B from Day 4. Horizontal bars in bHB and Insulin indicate statistically significant deviation between the placebo and DGA subgroups.

**FIGURE 5:**
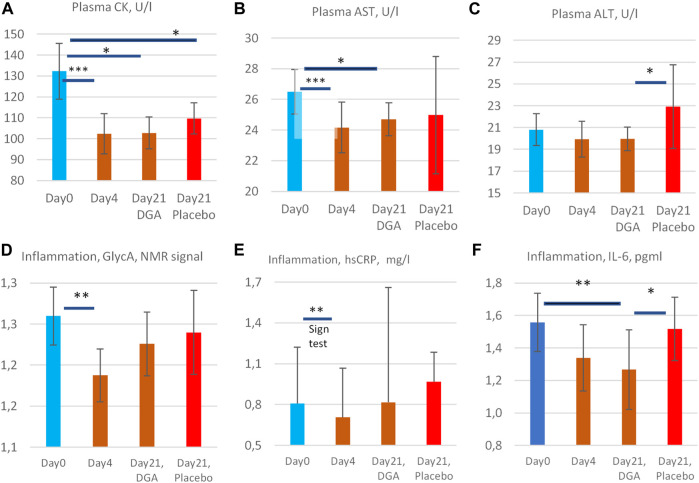
Enzyme release of creatine kinase **(A)**, aspartate aminotransferase **(B)**, and alanine aminotransferase **(C)** into the plasma, and low-grade inflammation markers GlycA **(D)**, hsCRP (**E)**, and IL-6 **(F)**. All statistical tests are based on intra-individual changes. SEM error bars are calculated from absolute values. Notes: 1) Day 21 bars in **(A–F)** are indexed to Day 0 bars so that the Day 21 results fully reflect the changes from the relevant DGA and placebo subgroups at Day 0. 2) There was one clear outlier in the 4-days result in CK and AST from the same participant (see Supplementary Presentation 1, Figures H,I), and one outlier in IL-6 Day 0 results (Supplementary Presentation 1, Figure P). Additionally, in GlycA (Figure 3D) the highest 1/3 ranked by VO_2_max deviated clearly from the other participants and from hsCRP (see Supplementary Presentation 1, Figures N,O). These observations have been excluded to achieve normality of the data. Importantly, even including these outliers, the respective changes in CK, AST, GlycA, and IL-6 would have been statistically significant. 3) The hsCRP data is non-normal due to very high volatility. Due to the high volatility median values of hsCRP are presented in **(E)** and a non-parametric Sign test was used for the 4-days change. 4) All other bars represent arithmetic means and the intraindividual changes for all others were normally disrtibuted. Statistically more powerful paired *t*-test was used when comparing the intraindividual changes from Day 0 in **(A,B,D, and F)**. 5) Naturally, when comparing the changes from Day 0 to Day 21 in the placebo group vs. the DGA group (ALT and IL-6, **(C,F)**), a paired *t*-test could not be used. Instead, a non-paired *t*-test was used to compare the changes in the placebo vs. DGA for ALT and IL-6.

The 4- and 21-days changes of carefully selected plasma metrics and mRNA expressions from WBCs were used as indirect primary markers for the activation of mitochondrial metabolism. Already after 4 days of the DGA regimen, there is a significant downregulation of all plasma energy substrates (Figure 2). Especially the strong decline in plasma lactate indicates an upregulation of the whole-body mitochondrial (energy) metabolism (Figure 3). In section @ADME and the Timing of the Response of the DGA Activation@, we shortly outline the ADME of DGA and how the response to the DGA regimen materializes from the acute single-dose towards the 4 days administration. The 21-days results show that the whole-body homeostasis has been restored but at a more “energetic” level, the liver and muscle functions having especially improved (Figure 4). Hepatic enhancement is visible from AST and ALT results and muscular improvements from CK results (Figure 5). Low-grade inflammatory markers IL-6 and GlycA were also our ex-ante-defined primary markers. Positive results on the reduction of chronic inflammation are presented in Figure 5 and related analyses with hsCRP as an additional proof of concept. Notably, CK, AST, GlycA, and hsCRP showed a strong positive response already to the 4-days DGA regimen (Figures 5A,B,D,E).

Day 0 starting values and the 4 days %-changes of all used plasma metrics are ranked by individual VO_2_max results and presented in Supplementary Presentation 1. Cellular level studies on WBCs and hepatocytes are reported in Supplementary Presentation 2, 3 respectively. Acute 45-min measurements on IL-6 and insulin are presented at the end of Supplementary Presentation 1 (“VO_2_max correlating markers2”) and in Supplementary Presentation 4. References are made to supplements when needed to support conclusions.

### Plasma Energy Substrate Responses to the 4-Days DGA Regimen

Plasma energy substrate concentrations reflect intracellular (cytosolic) concentrations via plasma membrane transporters (Figure 3). Main energy substrates like glucose, TGs, and FAs can be restructured and stored in cells and thus are not excreted back into the plasma. Smaller cytosolic metabolites like lactate and pyruvate can be efficiently imported or exported via plasma membrane monocarboxylic acid transporters (Figure 3).

Presented substrates in Figure 2A represent all main nutritional categories i.e., fats, carbohydrates, amino acids, ketone bodies, and carboxylic acids. They cover some 90% of used energy substrates at rest. All plasma concentrations of these energy substrates were downregulated by the 4 days DGA regimen (Figure 2A). Observed consistent response extremely likely reflected a decrease in intracellular concentrations of the substrates because the other possibility of increased excretion out of the body from plasma due to the DGA regimen can be sufficiently ruled out by careful organ and substrate-level assessments (Figures 2A,B).

Intracellular energy substrate concentrations may decrease through metabolism into either the TCA (Martínez-Reyes and Chandel, 2020), into anabolic reactions, or into intracellular energy stores. All these three metabolic alternatives relate somehow to mitochondrial metabolism because most of the anabolic reactions like gluco- and glyceroneogenesis materialize via mitochondria (Figure 3). Also, FA oxidation materializes in mitochondria.

#### Energy Substrate Net Inflow Into Cells Point to Mitochondrial Activation and Increased NADH/NAD^+^ -Ratio

We found that the 4-days DGA regimen caused an extremely significant 21% average decline in plasma lactate (*p* = 0.0009, Figure 2A). There does not seem to be any deviating persons in lactate responses despite the wide starting range of lactate concentrations (Supplementary Presentation 1, Figure A). When lactate is imported to the cells, its intracellular metabolic route is always via LDH and pyruvate towards mitochondria (Figure 3). In fact, all presented energy substrates eventually end in mitochondrial metabolism (when oxygen is present). Thus, the results presented in Figure 2A were a strong indication of increased mitochondrial metabolism at the whole-body level. Furthermore, the final catabolism of all energy substrates into CO_2_ produces energy-containing NADH molecules. Most of them are produced by the TCA and used by the OXPHOS (Figure 3). All in all, our results show an increase in overall NADH (energy) level in cells via DGA activation. We performed an additional *in vitro* experiment with cultured hepatocytes. Indeed, NADH generation was significantly activated 3 h after the last DGA dose (Supplementary Presentation 3).

#### The 4-days DGA Regimen Seems to Cause Temporary Hepatic Lactate Shortage

The first four bars in Figure 2A represent energy substrates that are mostly provided by the maintaining organs (arrows in Figure 2B) and the last 5 bars (excl. the DGA and insulin) represent substrates that are mostly imported to the liver for gluco- and glyceroneogenesis (arrows in Figure 2B).

There is an interesting difference between the first 4 and next 5 bars in Figure 2B. Although all are down, the substrates going towards the liver are all statistically significantly or even extremely significantly downregulated, but none of the reductions in the substrates going towards peripheral tissues is significant. It seems that during the 4 days DGA activation a scarcity of gluco- and glyceroneogenic plasma substrates towards the liver had clearly developed. Furthermore, from the volume decreases (right-hand scale, horizontal bars in Figure 2A) we can clearly observe that the scarcity was almost fully due to a decline in lactate.

A statistically very significant 21% decline in plasma glycerol (Figure 2A) demonstrates lactate shortage for hepatic glyceroneogenesis. Glycerol is not a direct energy substrate and thus the DGA regimen should not have any major effect on it. Additionally, glycerol kinase enzymes are active mostly in the liver. A robust 21% decline in glycerol indicates that the liver started to import free glycerol from plasma to form glycerol phosphate by hepatic glycerol kinase reactions and to thusly compensate the sharp reduction of plasma lactate. Lactate is the main substrate for hepatic glyceroneogenesis and glycerol phosphate is its product (Nye et al., 2008). Further proof comes from the surprising but temporary decline in plasma glucose, Figure 2A which also points to a mild shortage of lactate. This time for hepatic gluconeogenesis at Day 4 (Figure 2A).

#### Insulin Hinders Glucose Intake to Tissues Likely Because of Increased Intracellular Glucose Storage due to the DGA Activation

At the whole-body level, the temporary and benign lactate shortage has likely developed due to increased glycogen (glucose) and TGs/FAs storage into peripheral tissues like skeletal muscles. Plasma glucose level declines statistically almost significantly despite less stimulation from insulin (Figure 2A). One credible explanation in resting measurements was that on top of the lactate shortage also glycogen storage in peripheral tissues was likely to have been increased, as also happens after physical exercise by skeletal muscles (Hermansen et al., 1970; Goodwin, 2010). Also, TGs and FFAs seemed to be used/stored at a slightly higher rate under the DGA regimen (Figures 2A, 4A) (Hargreaves and Spriet, 2020). More detailed analysis on plasma TGs and FFAs response to the 4-days DGA regimen is presented in Supplementary Presentation 1 (Figures K,M, and K and M clustered).

#### The 4-Days DGA Regimen Causes Strong Modulation of Mitochondria-Related mRNA Expressions in the Immune Cells

The mRNA expressions of certain ATP synthase genes were strongly downregulated at Day4 (Supplementary Presentation 2, Supplementary Presentation 1, Figures B,C). Cellular ATP production is tightly regulated to avoid excessive ATP production. Also, the mRNA expression of the PC gene (Figure 3) was upregulated statistically very significantly during the 4 days DGA regimen (Supplementary Presentation 2, Figure B). In mitochondria the PC enzyme directs incoming energy substrate flows towards energy-consuming anabolic directions (Connelly et al., 2017; Dierckx et al., 2018). Excessive NADH and ATP are the hallmarks of mitochondrial activation at rest, and in that kind of environment PC enzyme reaction is favored (Zhang et al., 2014). All in all, the oversupply of NADH energy for ATP production at Day4 (Figure 2A) seemed to have a noticeable effect also on the WBCs.

#### ADME and the Timing of the Response of the DGA Activation

It seems that the DGA does not accumulate into plasma or tissues, on the contrary. The decline in plasma DGA concentration in 4 days (−4.5%, Figure 2A) showed that all of the ingested DGA 12 h earlier had been fully metabolized from plasma into tissues. Consistently, the conversion of DGA into glycerate-2-phosphate had somewhat accelerated during the 4 days DGA administration. In line with this, the mRNA expression of mitochondrial and cytosolic GLYCTK enzymes increased in 4-days (Supplementary Presentation 2, Figure F).

As a small natural molecule, water dissolved DGA is very rapidly absorbed from the gastrointestinal tract into tissues. In the present experiment, already in 45 min after the last DGA dose (Figure 1C) the plasma concentration of DGA was at a level that was roughly the same as the 3.33 mg/BW kg dose indicates for average concentration in the whole-body (data not shown). This result demonstrates that the distribution to tissues was on average very rapid. The DGA dose used was equivalent to 32 μmol of DGA/BW kg, which is roughly 8 times the average normal DGA concentration in plasma (4.0 μmol/L at Day 0).

Supplementary Presentation 1 (Figures P,Q clustered) show that the acute DGA dose already possesses an observable effect on IL-6 and insulin within 45 min (Supplementary Presentation 1 (Figures P,Q clustered and Supplementary Presentation 4). In hepatocytes (*in vitro*) a strong effect on NAD+/NADH-ratio was observed 3 h after the last DGA dose (Supplementary Presentation 3). Measured 12 h effects after the last dose may thus be and often are based on more rapid reactions and numerous cumulative energy metabolic interactions thereafter.

### The 21- and 14-Days Responses to the DGA Regimen

At Day 21 average glycerol concentration was back to the original level in the DGA group (Figure 4A). This shows that the mild hepatic lactate shortage at Day4 could be compensated in the longer term. Furthermore, during the 14 days follow-up period, the Day0 concentration in glycerol was reached more rapidly in the DGA group compared to the placebo (Figure 4B).

Also, glucose homeostasis was restored at Day 21, and furthermore faster in the DGA group compared to the placebo (Figure 4A). Interestingly, the response of insulin after the ending of the 4 days DGA regimen at Day 4 causes a clear deviation in the placebo group versus the DGA group (Figure 4B). The error bars in Figure 4B show that the volatility of the individual changes in insulin was too high for reliable quantitative analyses especially in the placebo group.

At Day 21, only lactate, pyruvate, and alanine continued to be statistically significantly downregulated in the DGA group. bHB was strongly upregulated (Figures 4A,B). These were clear signs of continued efficacy with the reduced ½DGA dose, as was expected. Reduction in alanine at both Day 4 and Day 21 in the DGA group likely showed continued hepatic energy substrate demand.

In Figures 4A,B bars represent changes in group averages and SEMs are calculated from individual %-changes (similarly to Figure 2A). The 21-day period was our primary target period ex-ante. In the present study, all participants were primed with the 4-days DGA regimen. Insulin data was very volatile but nevertheless, the changes from Day 4 to Day 21 were normally distributed. The parametric *t*-test between the placebo and DGA in insulin was statistically significant (Figure 4B) but the non-parametric Mann-Whitney *U*-test was not.

#### Strong Increase in bHB Points to Exercise Signaling

Plasma bHB increased 24% at Day 21 in the DGA group and there was no upregulation in the placebo (Figures 4A,B). bHB is a ketone body that is synthesized in the liver. It represents an essential carrier of energy from the liver to peripheral tissues, especially to the high energy demanding CNS tissues (Figure 2B) (Jensen et al., 2020). bHB concentration tends to increase during periods of prolonged exercise when the supply of glucose is too low for the energetic needs of the peripheral tissues (Newman and Verdin, 2017). An increase in plasma bHB in the DGA group at Day 21 pointed to the activation of the liver. Possibly this may have happened via some kind of repeated exercise signaling by the DGA regimen. bHB is the most abundant ketone body in mammals and functions also as an important signaling molecule that, e.g., suppresses inflammation and oxidative stress (Shimazu et al., 2013; Chriett et al., 2019). Interestingly at Day 4, it seemed that the demand of bHB from peripheral tissues exceeded the supply of bHB from the liver temporarily (Figure 2A).

#### Pyruvate at Day 21

Interestingly, at Day 21 the decline in plasma pyruvate (−22%) exceeded lactate (−19%) in the DGA group (Figure 4A). At Day 4, lactate was down 21% and pyruvate down “only” 18% (Figure 2A). When pyruvate is directly acquired from plasma for mitochondrial use, it lessens cytosolic NADH generation from inverse LDH reaction (Figure 3). The shift between lactate and pyruvate changes possibly indicates that intracellular NADH levels had reached some kind of “upper limit” already at Day 4.

Intracellular pyruvate may enter mitochondrial metabolism via PDH or PC enzymes (Figure 3). PC reaction does not produce NADH, CO_2_, and acetylCoA like the PDH enzyme reaction does; instead, it consumes one ATP and CO_2_ to form OAA (Figure 3). The PC enzyme reaction is favored in “over-energetic” situations like at Day4 (Berg et al., 2002; Zhang et al., 2014; Liu et al., 2018). Based on the above, it is easier to understand why plasma pyruvate surprisingly continued to be also be statistically significantly downregulated in the placebo subgroup at Day 21 (Figure 4A). In the test setup used, the extraordinarily strong 4-days effect of the DGA regimen on pyruvate seemed to have caused a more permanent remodeling of mitochondrial pyruvate demand that lasted until Day 21.

### Moderate Activation of Overall Mitochondrial Metabolism Reduced Enzyme Release of CK and AST to Plasma as Well as Low-Grade Inflammation

Next we measured health parameters indirectly related to mitochondrial activation

#### Increased NADH, ATP, and Substrate Availability Likely Enhanced Cellular Membrane Integrity

Plasma CK, an indicator of mitochondrial and plasma membrane health status in skeletal and cardiac muscles and other high energy demand tissues (Rojo et al., 1991; Schlattner et al., 2006), AST, an indicator of hepatic and overall cell membranes health status (Hall and Cash, 2012; Gączarzewicz et al., 2015), and ALT (plasma membrane health status mainly in the liver) (Hall and Cash, 2012) were reduced during the DGA regimen (Figures 5A–C). The reduction of CK (−23%) and AST (−9%) were statistically extremely significant during the 4-days DGA regimen (Figures 5A,B).

The surprisingly rapid and consistent decline in CK and AST was fully in line with mitochondrial activation that increases NADH and ATP supply for anabolic cell membrane maintenance reactions (Hall and Cash, 2012; Dias and Nylandsted, 2021; Kim et al., 2019). Interestingly, energy metabolic activation requires enhanced assembly and maintenance, especially of mitochondrial membranes. The slower reaction of plasma ALT (Figure 5C) may signal that plasma membrane integrities were not so acutely enhanced (Ramos et al., 2013). ALT enzymes are present only in the cytosol but CK and AST enzymes both possess cytosolic and mitochondrial subtypes.

In the placebo group, the 4-days DGA regimen produced a noticeable continuing effect in CK and AST at Day 21 (Figures 5A,B). CK remained even statistically significantly downregulated in the placebo at Day 21, in line with pyruvate earlier. It seems that the 4-days DGA regimen possesses much longer effects for certain matrices than was expected before the study. Case CK indicates that the tissue source for sustained DGA effects may be muscle mitochondria (Rojo et al., 1991; Schlattner et al., 2006), but more research is needed.

#### Low-Grade Inflammation Was Reduced Both in 4- and in 21-Days

We measured low-grade inflammation by 3 different indicators GlycA, hsCRP, and IL-6. All showed that low-grade inflammation declined surprisingly fast in 4-days and remained lowered in 21-days compared to Day 0 starting values (Figures 5D–F). GlycA and IL-6 were our primary markers for low-grade inflammation but hsCRP was also measured for confirmation. Observed effects were as expected but the reduction in subclinical inflammation turned out to be even stronger than we anticipated.

GlycA is a novel sensitive biomarker of subclinical inflammation that correlates well with hsCRP but may be more sensitive and stable in disclosing systemic inflammation (Connelly et al., 2017; Dierckx et al., 2018). Due to high volatility, the hsCRP results deviated from the normal distribution, and median hsCRP was used instead of the mean (Figure 5E). Based on the non-parametric SIGN test, the observed −13% decline of the median hsCRP was statistically very significant under the 4-days DGA regimen. Remarkably almost all individual hsCRP values were reduced in 4-days (Supplementary Presentation 1, Figure N).

IL-6 possesses multiple effects. Chronically elevated IL-6 is a general sign of elevated sub-clinical inflammation although the tissue source of IL-6 may be relevant (Han et al., 2020). There is evidence that a long-term physical activity regimen lowers IL-6 in aging persons and that the reduction is stronger than in CRP (Nicklas et al., 2008). Additionally, an acute increase in plasma IL-6 causes adipose tissues to release FFAs to plasma (van Hall et al., 2003; Brown et al., 2015). This may happen, e.g., after a strenuous VO_2_max test. Acute IL-6 response is analyzed in Supplementary Presentation 4.

In the longer-run pro- and anti-inflammatory aspects of IL-6 release dominate its metabolic effects. Based on Figure 5F, we can conclude the statistically very significant, roughly 20% reduction of plasma IL-6 at Day 21 was a strong indication of lowered subclinical inflammation in the studied 50–60-year-old apparently healthy persons.

There is evidence that endogenous mitochondrial activation in the whole-body, e.g., an increase in physical activity, is able to reduce inflammation (Nicklas et al., 2008). Also in our study model, the DGA regimen caused an overall mitochondrial activation that seemed to lead to an enhancement in both systemic (WBCs) and in local level immune defenses. Local-level enhancement materializes in the main organs and metabolically active tissues. The root causes of these cellular-level improvements are based on enhanced mitochondrial energy metabolism as presented in the **Graphical Abstract** (Olek et al., 2004; Handschin and Spiegelman, 2008; Camara et al., 2010; Li et al., 2013).

## Conclusion

Extremely solid results were obtained on lactate in the 4-days DGA regimen in the present study. Practically all excess lactate in all participants was imported from plasma into tissues. Combined with other results, this was a sign of surprising whole-body mitochondrial activation. Skeletal muscles and other energy metabolically active peripheral tissues were already affected in 4-days. Furthermore, biomarkers for hepatic activation were positively affected, e.g., the changes in plasma bHB, alanine, and ALT deviated from the placebo at Day21. An increase in plasma bHB forms an important energy supply to neuronal tissues. Exogenous DGA regimen additionally activated the mRNA expressions of mitochondrial metabolism in circulating immune cells. Robust reductions in CK and AST point to enhanced cellular membrane integrity already in 4-days and the improvement lasted until Day 21. Furthermore, subclinical inflammation was reduced already after the 4-days DGA regimen, and it remained lowered at Day 21.

Because the amounts of administered DGA were micromolar and cannot directly have caused observed millimolar and other significant changes, we hypothesize that an increase in tissue and cellular DGA concentration causes an unknown indirect signal or a cascade of intracellular signals: “more aerobic energy is needed”. We call it the DGA activation. It seems to cause high energy NADH concentration for ATP production to rise intracellularly. When extra ATP is not needed, reduced NADH molecules may be used in anabolic reactions and in the neutralization of oxidized molecules. All these processes re-oxidize NADH into NAD^+^ that is essential for maintaining cellular energy metabolism both in the mitochondrial matrix and in the cytosol. In the present experiment, it seemed that the extra reducing power from NADH was dominantly “invested” into continuously ongoing cellular maintenance reactions.

Our experiment was restricted to participants with normal weight. Clearly overweight or underweight persons may need a different kind of administration, or all the strong effects may not be valid in these groups or otherwise in non-healthy subgroups. Another limitation was that the 4-days effects of DGA carried on much longer than we expected when planning the study. Surprisingly, pyruvate and CK continued to be statistically downregulated even after 14-days in the placebo. This was not expected and partially challenged our study model. In future studies, the follow-up period with the placebo group should be at least 3–4 weeks or to have totally separated blinded placebo and treatment groups.

Based on the obtained results, mitochondrial metabolism seemed to be consistently but gently upregulated by the DGA activation. No adverse effects were reported during or after the study despite specific questionnaires. Future research should be directed to both short- and long-term effects of the DGA activation. Acutely it may facilitate recovery from physical exercises and some acute metabolic challenges, like infections. It may also alleviate chronic energy metabolic disorders in main organs, like the liver, CNS, and muscles. Enhanced membrane integrity combined with lower systemic inflammation and activated metabolic flows may be especially beneficial for the aging population.

## Data Availability

The original contributions presented in the study are included in the article/Supplementary Material, further inquiries can be directed to the corresponding author.

## Footnotes

1 One person had to be fully excluded from the final results because of 10 days prior he had initiated a low-calorie diet. Another person was excluded from the Day4 results because of reported significant work stress the previous night.

## Abbreviation

ADME, absorption, distribution, metabolism, and excretion; ALT, alanine aminotransferase; AST, aspartate aminotransferase; ATP, adenosine triphosphate; bHB, beta-hydroxybutyric acid; CK, creatine kinase; DGA, D-glyceric acid; FA oxid, fatty acid oxidation; FAs, fatty acids; FATP, fatty acid transport protein; FFAs, free fatty acids (Total FAs–TGs); GlycA, glycoprotein acetyls; GLUT, glucose transporter; IL-6, interleukin 6; JyU, University of Jyväskylä; LDH, lactate dehydrogenase; MCT, monocarboxylic acid transporter; NADH, reduced nicotinamide adenosine dinucleotide; NAD+, oxidized form of NADH; OAA, oxaloacetate; OXPHOS, oxidative phosphorylation; PC, pyruvate carboxylase; PDH, pyruvate dehydrogenase; PEP, phosphoenolpyruvate Enzymes; PK, Pyruvate kinase; TCA, tricarboxylic acid cycle; TGs, triglycerides.
