# Supplementary material for: Randomized Trial: D-Glyceric Acid Activates Mitochondrial Metabolism in 50–60-Year-Old Healthy Humans
Source: Front Aging. 2021 Oct 29;2:752636. doi: 10.3389/fragi.2021.752636 (PMC9261421; doi:10.3389/fragi.2021.752636)
Supplement: Supplementary file 1 [file Presentation1.pdf]

Presentation 1 / Supplement A

Day0 concentrations and %-changes from Day0 to Day4 ranked by VO_2max

SAFigures A, B, C, D, E, F, G, H, I, J, K, L, M, N, O, P and Q. Day0 individual starting concentrations (dotted-lines, righthand scale) and the 4 day %-changes under the DGA regimen (bars, lefthand scle). Lactate (A), pyruvate (B), glucose (C), glycerol (D), alanine (E), citrate (F), bHB (G), CK (H), AST (I), ALT (J), TGs (K), BMI (L), FFAs (M), hsCRP (N), GlycA (P), IL-6 (P) and insulin (Q). Data is ranked individually from the highest to the lowest VO_2max at Day0.

Additional graphs with data clustering based on two VO_2max subgroups combined with short analyses has been made for TGs, FFAs, GlycA, hsCRP, IL-6 and insulin. Outliers did not exist in practice, but one outlier related to CK/AST and another related to IL-6 are explained below relevant figures, as well as heteroscedastic subgroup devition in GlycA (SAFig.O and O clustered).

Plasma energy substrates that did not correlate with individual Day0 VO_2max result

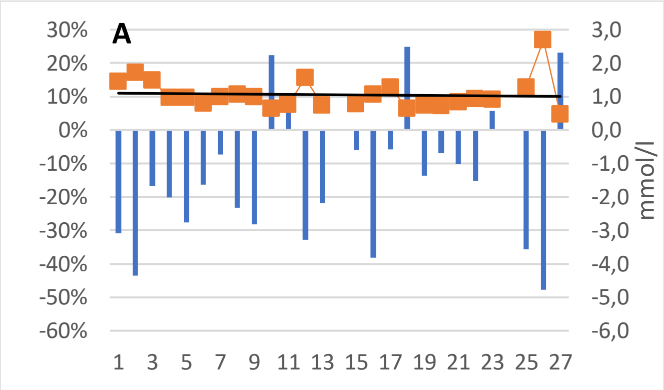

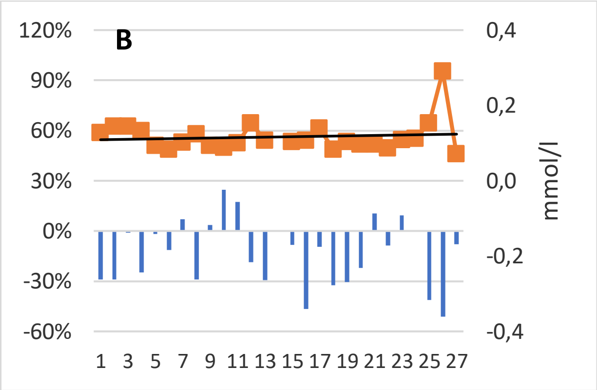

Four out of five participants whose lactate concentration did not decline (bars in SAFig.A), possessed exceptionally low starting lactate concentration (dot-line). Reason for this may be that some tissues use lactate e.g. during exercise [47].

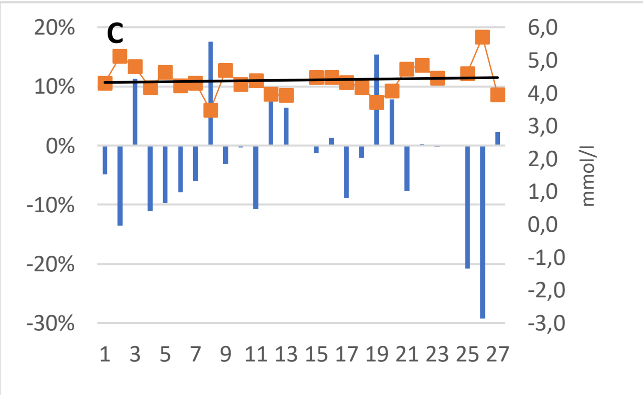

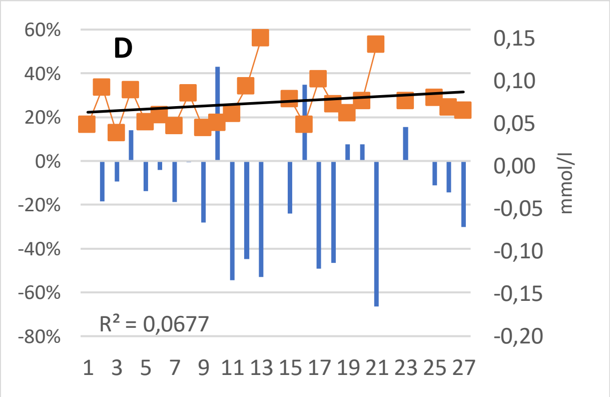

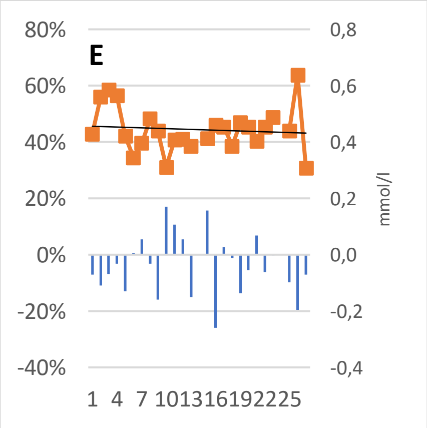

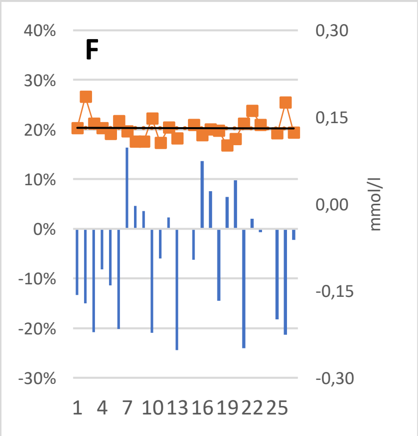

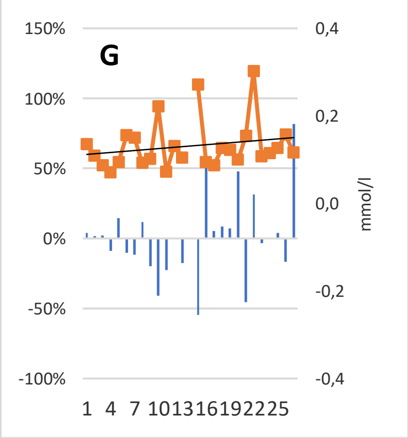

Plasma Enzyme Release: CK (**H**), AST (**I**) and ALT (**J**) and An Outlier Analyses

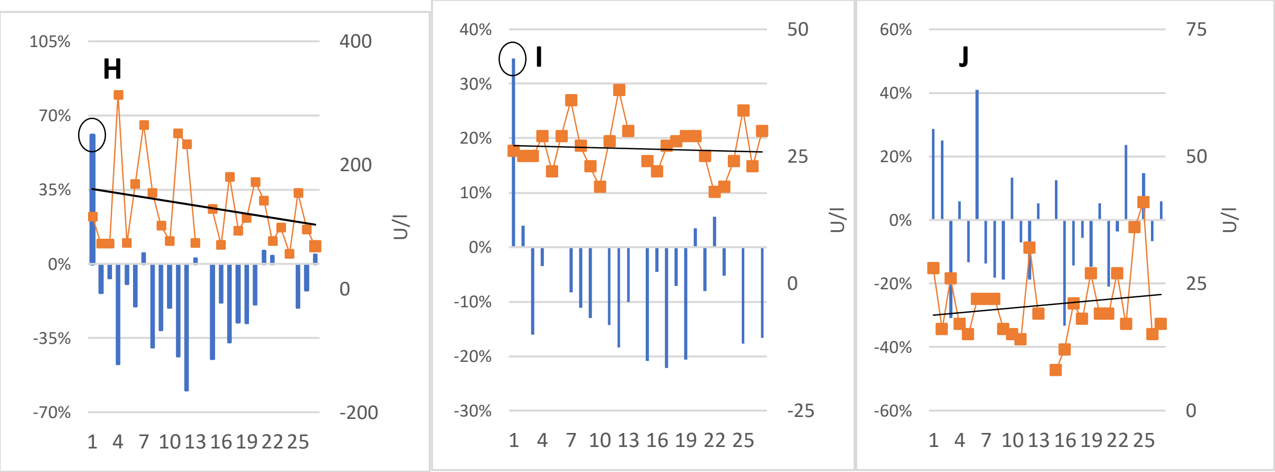

OUTLIER ANALYSES: One participant possessed extremely deviating changes from others for both CK (+61%, avg. -23%) and AST (+35%, avg. -9%) at Day4 (SAFig.H and I observation nro 1). All other 4-day results were in line with others and at Day21 the change from Day0 of this participant was fully in line with other group members (data not shown). We can conclude that the Day4 observation was an outlier not caused by the 4-day DGA regimen. Because the liver specific plasma ALT was in line with other participants (SAFig.J), we may cautiously conclude that the outlier in CK&AST was likely caused by some small and temporary muscular trauma [23b]. The outlier was excluded from results in Fig.5 A and B to achieve normality.

VO$_2$max correlating markers1: TGs, BMI, FFAs, and GlycA, and hsCRP as a comparison for GlycA

Some exceptional metabolites / metrices correlated rather strongly with the individual VO$_2$max results at Day0. These Day0 correlations were naturally independent of the DGA regimen. Nevertheless, when there exists a very clear difference between higher VO$_2$max and lower VO$_2$max participants at Day0, also the 4-day DGA activation might possess different effects VO$_2$max subgroups (SAFigs. K, M, and O). There may be several reasons. One relates to the hypothesized mechanism of action of DGA. VO$_2$max directly reflects each individual's aerobic capacity, which is essentially the same as persons' overall mitochondrial capacity. Our primary hypothesis is that the DGA activation causes an overall activation in mitochondrial metabolism, thus in some cases it is even expected that there is a difference between VO$_2$max subgroups.

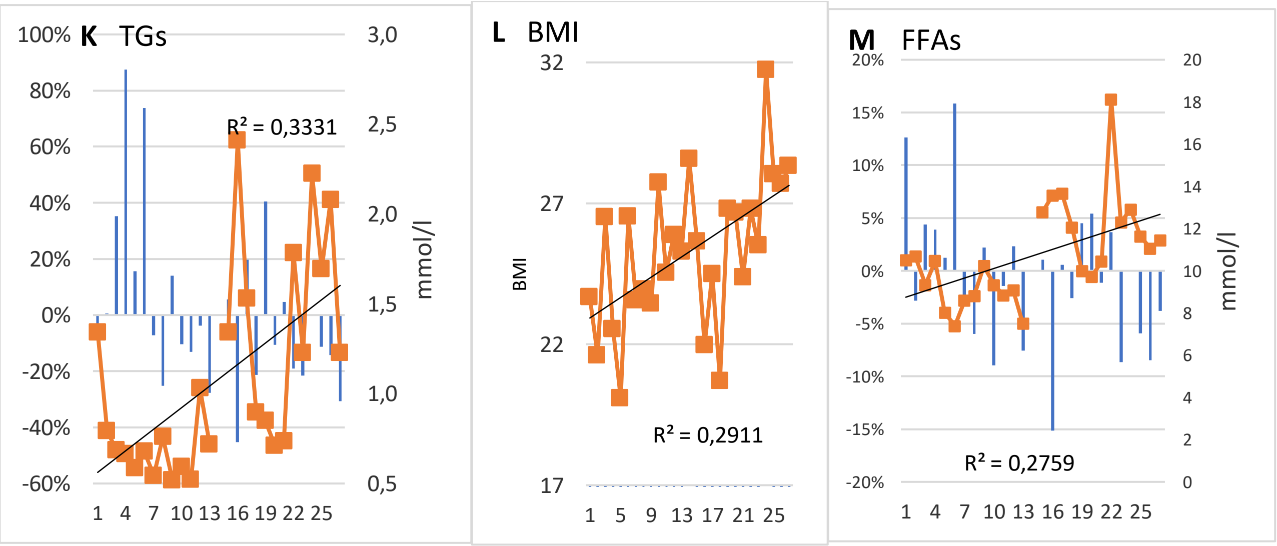

Clustering the HC_1/3 and MID&LC_2/3 subgroups

To identify and analyze possible VO_2max effects consistently, we formed two non-overlapping subgroups with N=9 and N=2x9 participants in each covering all data. Nine participants with the highest VO_2max result at Day0 form the "HC_1/3 subgroup" and 18 participants with lower VO_2max at Day0 form the "MID&LC_2/3 subgroup". The DGA results independent basis for this subgroup clustering can be derived from Supplement D, in which we show that 30 minutes after the VO_2max cycling the HC_1/3 subgroup differs from the MID&LC_2/3 subgroup. The 4-day results (SAFigs. K/TGs, M/FFAs, and O/GlycA) and the 45 minutes results (SAFigs. P/IL-6 and Q/insulin) based on this clustering are presented below, always after the relevant graph with individual values.

TGs and FFAs clustering

Figures below (Figs. K and M clustered) explain partially why the overall impact of the DGA activation on TGs and FFAs was subdued in the whole group (Fig.2A) compared to many other metrics. There was an effect on FAs, but in both the TGs and FFAs there existed statistically significant difference between the HC_1/3 and MID&LC_2/3 subgroups. (Comparison based on parametric t-tests of 4-day %-changes.)

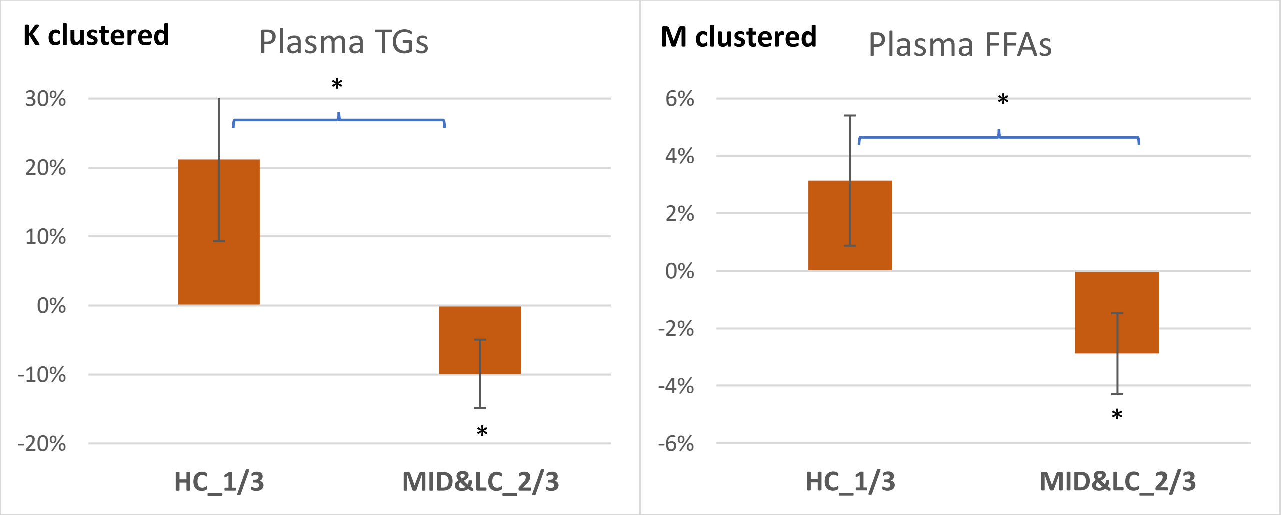

It is well known that aerobic exercise training enhances fatty acid use and storage in skeletal muscles [24]. In the present study group, this fact is directly indicated by lower initial plasma lipid concentrations in more trained persons (SAFigs. K and M). Additionally, the HC_1/3 participants are physically better trained than MID&LC_2/3 participants and thus the capacity to replenish energy stores after physical exercises should also be better. It is likely that the DGA activation caused an inflow of FAs in all participants but in the HC_1/3 subgroup hepatic release of TGs and adipose tissues release of FFAs managed to exceed increase demand by the peripheral tissues (Fig.2B). Supplement C shows that the DGA regimen is able to directly activate hepatic energy metabolism in vitro. Supplement D shows that plasma IL-6 is acutely increased in the HC_1/3 subgroup 45 minutes after DGA dose.

Especially in TGs the difference between the HC_1/3 and MID&LC_2/3 in the 4-day changes was very wide (SAFig.K clustered). Average plasma TGs at Day0 were 0.73 mmol/l (HC_1/3) and 1.27 mmol/l (MID&LC_2/3). Thus, the 20% increase in the HC_1/3 subgroup and the 10% decrease in the MID&LC_2/3 subgroup were both towards 1 mmol/l that can be considered more balanced TGs concentration.

GlycA and hsCRP

VO_2max correlation was strongest with GlycA (SAFig.O). Even though there are genetic differences in persons VO_2max it is generally known that exercise training increases aerobic capacity. Also, the tendency

of low-grade inflammation to improve with increased physical exercises is generally recognized. Kujala et al. have presented the association between VO_2max and glycoprotein acetyls (GlycA) in young males [48].

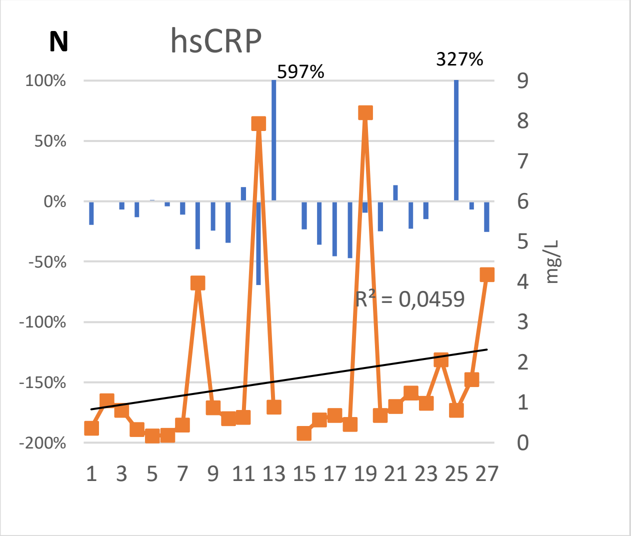

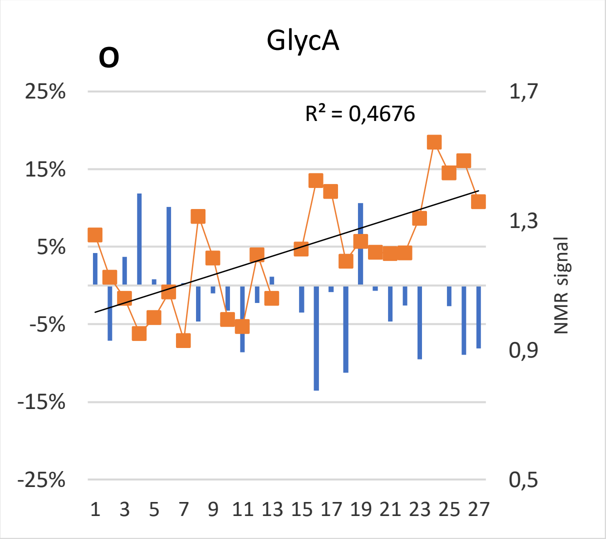

Clustered hsCRP and GlycA data comparison

In comparison to hsCRP and IL-6, GlycA was an excellent low-grade inflammation indicator for the MID&LC_2/3 sugroup (Fig.5D). There was nevertheless a surprising challenge in certain GlycA results in our study model. The problem relates only to the HC_1/3 subgroup and it becomes visible when we compare GlycA results to "identical" hsCRP below (SAFigs.N-O clustered). hsCRP points to statistically significant decline in low-grade inflammation in both the HC_1/3 and MID&LC_1/3 subgroups (SAFig.N clustered, paired t-tests were used). Additionally, at the whole group level all indicators (IL-6, GlycA, and hsCRP) pointed to reduced inflammation. Nevertheless, GlycA in the HC_1/3 subgroup deviates from all (SAFig.O clustered).

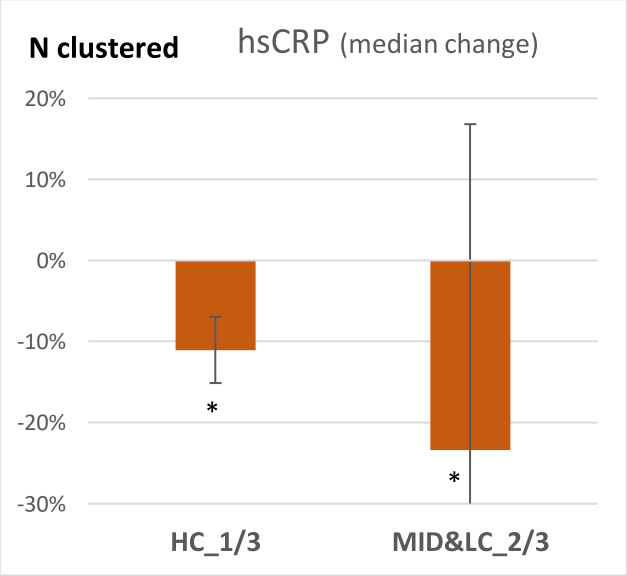

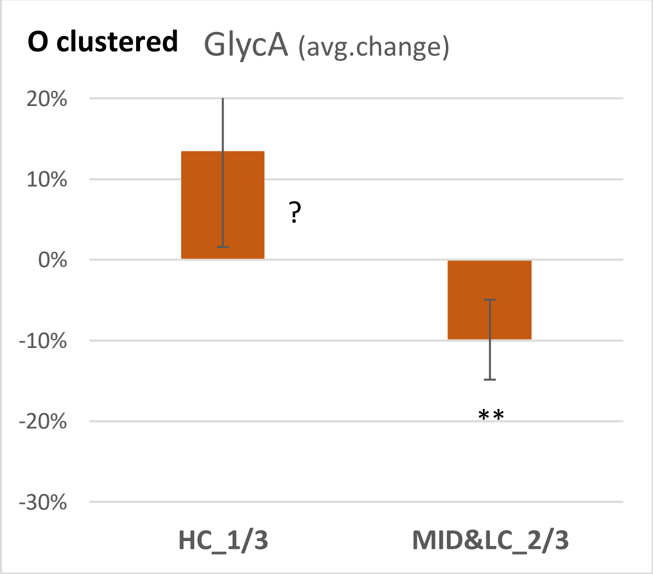

Because GlycA consists mostly of hepatic acute phase proteins and because in our study model there seems to be a temporary shortage of lactate for hepatic anabolic reactions (Section 3.1.2.), it may well be that some of the acute phase proteins give an exaggerated signal on inflammation in the HC_1/3 subgroup. Furthermore, the acute insulin response (SAFig.Q clustered) point to the fact that hepatic lactate shortage may be especially strong in the HC_1/3 subgroup. The deviation of the HC_1/3 subgroup GlycA was not

statistically significant and does not effect any of our conclusions, but it is an interesting observation that may be worth more research in similar test setting with other substances in the future.

VO_2max correlating markers2

IL-6 and Insulin

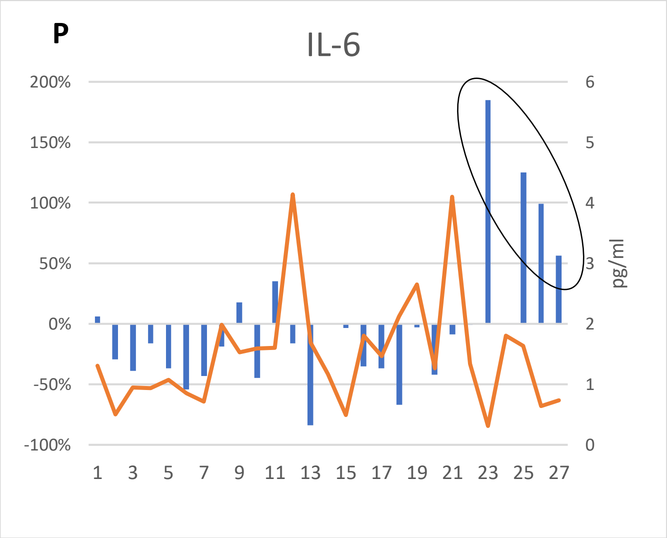

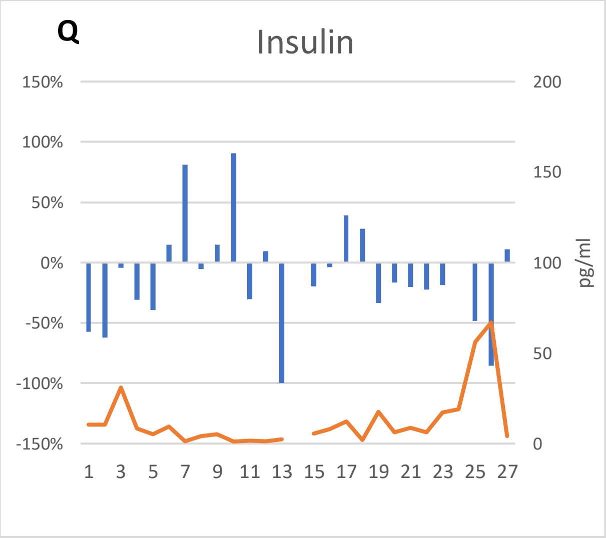

Most of the changes in IL-6 and insulin 12 h after the last DGA dose are negative at Day4 (SAFigs.P-Q). In IL-6 five lowest capacity persons tend to possess extraordinarily high increases at Day4 (SAFig.P). These extraordinary high increases fade away at Day21. (Only one observation, rank 23rd, remained exceptionally high +317%. It was ruled out as an outlier in the results presented in Fig.5F). Also, in insulin there was possibly a mild VO_2max dependency observable after 12 h (SAFig.Q).

Clustering of IL-6 and insulin acute 45 min responses

At 45 minutes the VO_2max dependent differences were nevertheless significant in both IL-6 and insulin. Like in the cases of GlycA, TGs, FFAs, the HC_1/3 subgroup deviates very clearly from the rest of the participants with lower initial aerobic capacity (SAFigs.P-Q clustered, paired t-tests were used in intra-subgroup tests and non-parametric Mann-Whitney U-tests were used in inter subgroup comparisons).

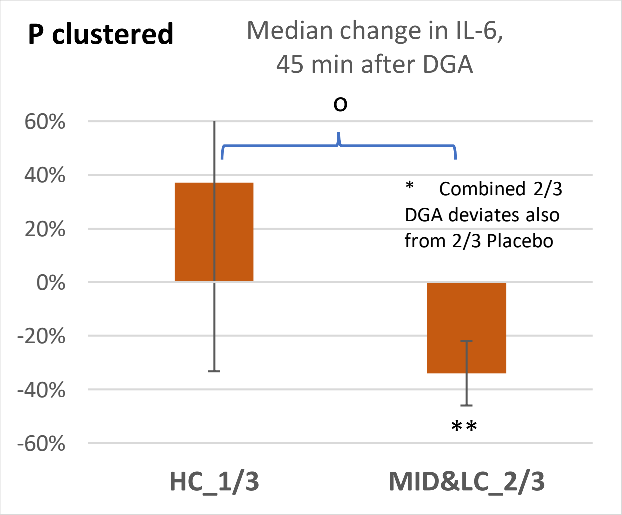

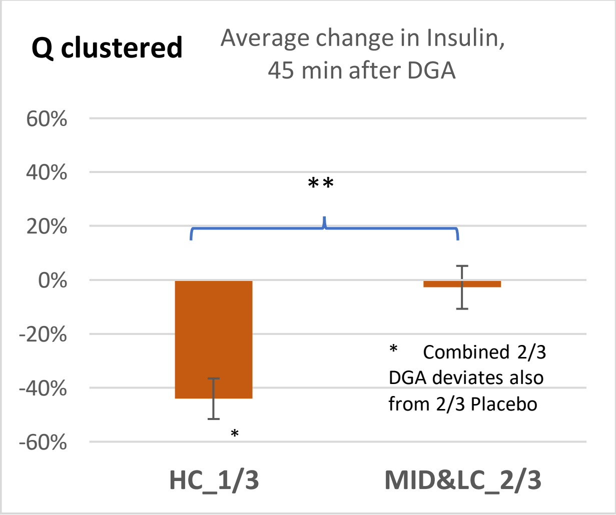

Interestingly, the MID&LC_2/3 group deviate from the same group placebo participants statistically significantly in both IL-6 and insulin. Thus, there existed an acute 45 min DGA effect in both.

In Supplement D we discuss the acute response of IL-6 after the VO_2max and the acute DGA dose. Thus, in here we elaborate only the acute effects on clustered insulin (SAFig.Q clustered). In our study model, plasma insulin directly reflects the difference between gluconeogenic organ's ability to supply glucose to peripheral tissues and the demand for plasma glucose from peripheral tissues (Fig.2B). When the glucose demand exceeds the supply, insulin is lowered to keep blood glucose stable by reducing the demand for glucose. (In the present study model insulin is clearly lowered when fasting is extended by 45 minutes, e.g. in the placebo insulin declined 25% in 45 minutes, data not shown.) In the MID&LC_2/3 subgroup the insulin decline was practically zero and it deviated from the placebo statistically significantly (SAFig.Q clustered). Thus, it seems that the acute DGA dose managed to activate gluconeogenic organs to supply glucose to compensate for extended fasting in the MID&LC_2/3 subgroup.

Conversely in the HC_1/3 subgroup, there was a deviating insulin response (SAFig.Q clustered). Observed robust decline likely indicates, on top of the fasting effect, that the glucose demand from muscles temporarily exceed the supply gluconeogenic organs in the HC_1/3 subgroup. Muscles aerobic capacity was clearly the main differing factor between the HC_1/3 and MID&LC_2/3 subgroups.
