# Supplementary material for: Randomized Trial: D-Glyceric Acid Activates Mitochondrial Metabolism in 50–60-Year-Old Healthy Humans
Source: Front Aging. 2021 Oct 29;2:752636. doi: 10.3389/fragi.2021.752636 (PMC9261421; doi:10.3389/fragi.2021.752636)
Supplement: Supplementary file 2 [file Presentation4.pdf]

Presentation 4 / Supplement D

Acute IL-6 response after the VO_2max test and after the acute DGA dose

All 12 hours earlier taken DGA is metabolized from plasma next morning (Fig.2A). Due to rapid metabolism, the repeated effects of the latest DGA dose generate very significant part of the 4- and 21-day effects. Below presented acute effects compared to VO_2max were secondary targets of the study because of huge challenges related to such a short timeframe. (Short 45-minute comparison period was primarily chosen for analyses of ADME and possible signaling effects of DGA after a bout of exercises of DGA.)

IL-6 was one of the primary biomarkers in the present study. It is generally known that IL-6 release from skeletal muscles is significantly increased after exercises. Further, that an acute increase in plasma IL-6 causes adipose tissues to release FFAs to plasma (van Hall et al., 2003) and thereby expedite evolutionarily important replenishing of muscular energy stores. Additionally, there exists a consensus that chronically elevated IL-6 is a sign of elevated sub-clinical inflammation and that it possesses a worsening effect on insulin resistance (Brown et al, 2015).

Fully in line with our expectations the acute VO_2max effect on IL-6 was statistically very significant for all after 30 min ending the VO_2max (SDFig.1A). Rather surprisingly, the magnitude of the effect of VO_2max test on IL-6 was clearly dependent on participants initial VO_2max (SDFig.1B), i.e., dependent on muscular mitochondrial aerobic capacity. The pattern on increased IL-6 release was most visible in the highest third ranked by VO2max (SDFig.1C). Average VO_2max for each subgroup is presented on the lowest line of SDFig.1C Furthermore, the HC_1/3 subgroup deviated from combined MID&LC_2/3 subgroup statistically very significantly (non-parametric Mann-Whitney U-test was used).

SDFigure 1. IL-6 30 minutes after VO2max ("Post") vs. before VO2max ("Pre"), total time frame 45 minutes for all. Paired t-test was used in SDFig.1A. In SDFig.1C, paired t-tests were used for intra-subgroup tests (HC_1/3 and LC_1/3) and a non-parametric Mann-Whitney U-test was used in inter subgroup comparison between HC_1/3 compared to combined MID&LC_2/3 subgroup.

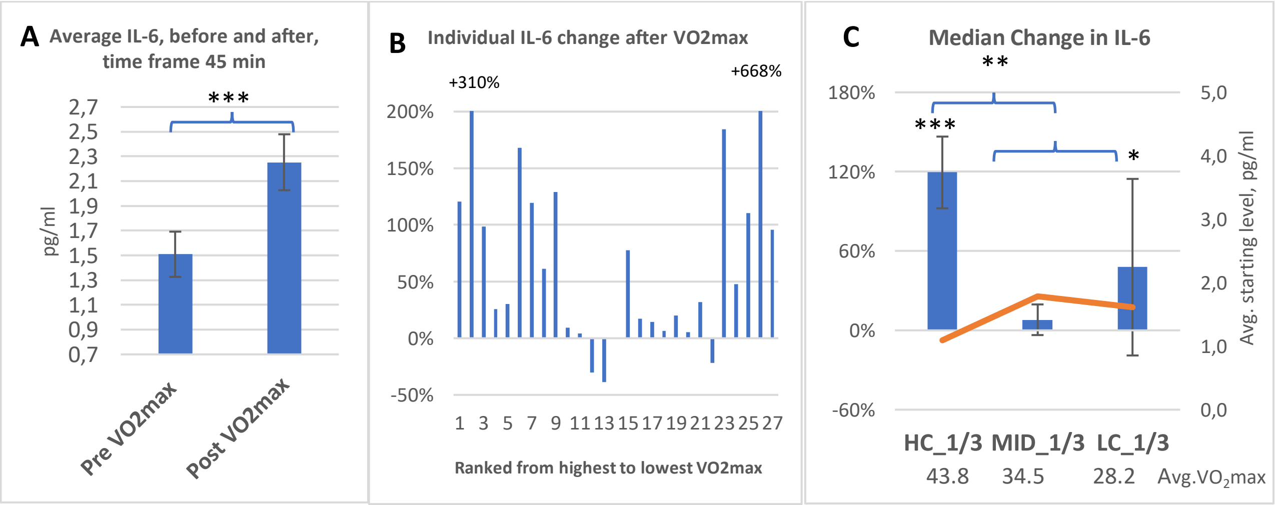

The pattern in IL-6 was similar 45 minutes after the acute DGA dose compared to VO2max (SDFig.2C). Furthermore, the IL-6 response in the HC_1/3 subgroup deviates statistically significantly from combined MID&LC_2/3 subgroup 45 minutes after the acute DGA dose. (At Day4 there had been already 8 x 12h DGA priming but from Fig.2A we can observe that the earlier doses of DGA have been fully metabolized from plasma.)

SDFigure 2. Plasma IL-6, before and after DGA or placebo at Day4 morning, total time frame 45 minutes for all. In SDFig.2C, paired t-tests were used for "combined MID&LC_2/3 subgroup compared to zero control" and for inter subgroup comparison between "combined MID&LC_2/3 subgroup compared to placebo" (the latter t-test was not justified by normality, but it is nevertheless indicative). Non-parametric Mann-Whitney U-test was used in inter subgroup comparison between HC_1/3 compared to combined MID&LC_2/3 subgroup in SDFig.2C

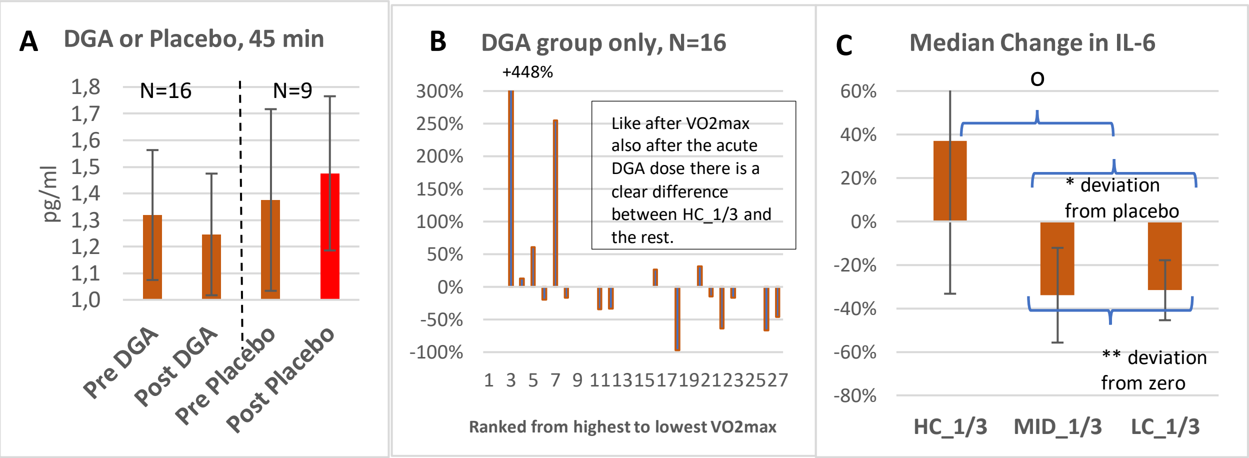

When we assume that the DGA effect ("the DGA activation") is closely linked to the mitochondria it is in fact rather natural that the most rapid effect can be seen only in the HC_1/3 subgroup because their aerobic / mitochondrial capacity is the highest. (VO_2max was 43.8 in the HC_1/3 compared to 31.35 in the combined MID&LC_2/3 subgroup.)

In our study plan, we were expecting a small acute increase in IL-6. Now it seems that the expected "exercise effect" of DGA via IL-6 materializes only in the HC_1/3 subgroup. In the MID&LC_2/3 subgroup the effect seems to materialize only gradually during 10-12 h of full metabolism of DGA. Furthermore, the anti-inflammatory effects of the DGA activation (Figs.5D-E) seem to overcome the IL-6 upregulating exercise effect already in 45 min in the MID&LC_2/3 subgroup and even statistically very significantly (SDFig.2C).

Most important take away from the above analyses is that the IL-6 response in the HC_1/3 subgroup was clearly faster both 30 min after ending the VO_2max cycling and 45 min after last DGA dose. Acute increase in IL-6 facilitates lipolysis in adipose tissues that increases plasma FFA concentration, which facilitates peripheral tissues, especially muscles, energy stores replenishing (Fig.2B). Described differences in acute IL-6 increase may explain part of the difference between the HC_1/3 and the MID&LC_2/3 subgroups in plasma FFAs. In Supplement A, we show that plasma FFAs possess similar VO_2max subgroup "distribution" that IL-6 albeit the time frame is shorter in IL-6 compared to FFAs (SAFig.M clustered).
