# Supplementary material for: Randomized Trial: D-Glyceric Acid Activates Mitochondrial Metabolism in 50–60-Year-Old Healthy Humans
Source: Front Aging. 2021 Oct 29;2:752636. doi: 10.3389/fragi.2021.752636 (PMC9261421; doi:10.3389/fragi.2021.752636)
Supplement: Supplementary file 4 [file Presentation2.pdf]

Presentation 2 / Supplement B

mRNA signal of Some Key Mitochondrial Enzymes in WBCs

The mRNA expressions of genes do not necessarily give direct information on the activity of the underlying enzymes or related metabolic pathways. Especially for the main metabolic pathways post transcriptional activations or deactivations by phosphorylation, acetylation etc. often influences real enzyme activities significantly more than the changes in the mRNA expression of even rate limiting enzymes. Additionally, excessive initial capacity of some enzymes at Day0 and/or cellular energy substrate stores may dampen possible mRNA responses significantly. Furthermore, in the study the mRNAs were measured from WBCs and sometimes their gene expressions do not represent tissues in general. Nevertheless, certain significant changes in plasma metabolites like seen in Figs. 2A and 3A-B were reflected in mRNA expressions.

SBFigures A-E. **mRNA expressions of Mitochondria Related Genes** (unit mRNA signal in all). In the mitochondria pyruvate carboxylase (PC) mRNA expression was activated very significantly under the 4-day DGA regimen and it returned to Day0 levels in both the placebo and DGA groups at Day21 (**A**). ATP synthase enzymes were significantly modulated at Day4 (**B-D**). Only MT-ATP8, an OXPHOS enzyme, was upregulated at Day21 in the DGA group (**B**) whereas other mRNAs returned to their Day0 levels after strong modulation at Day4 (**A-D**). Furthermore, all of the mitochondrially encoded mRNAs of OXPHOS genes were upregulated in the DGA group, but not in the placebo (**E**). GLYCTK mRNA was up regulated in 4-days and downregulated in the placebo at Day21 (**F**). More information on used statistical tests and SEM error bars, see below the graph and the explanations below Figure 5A-F.

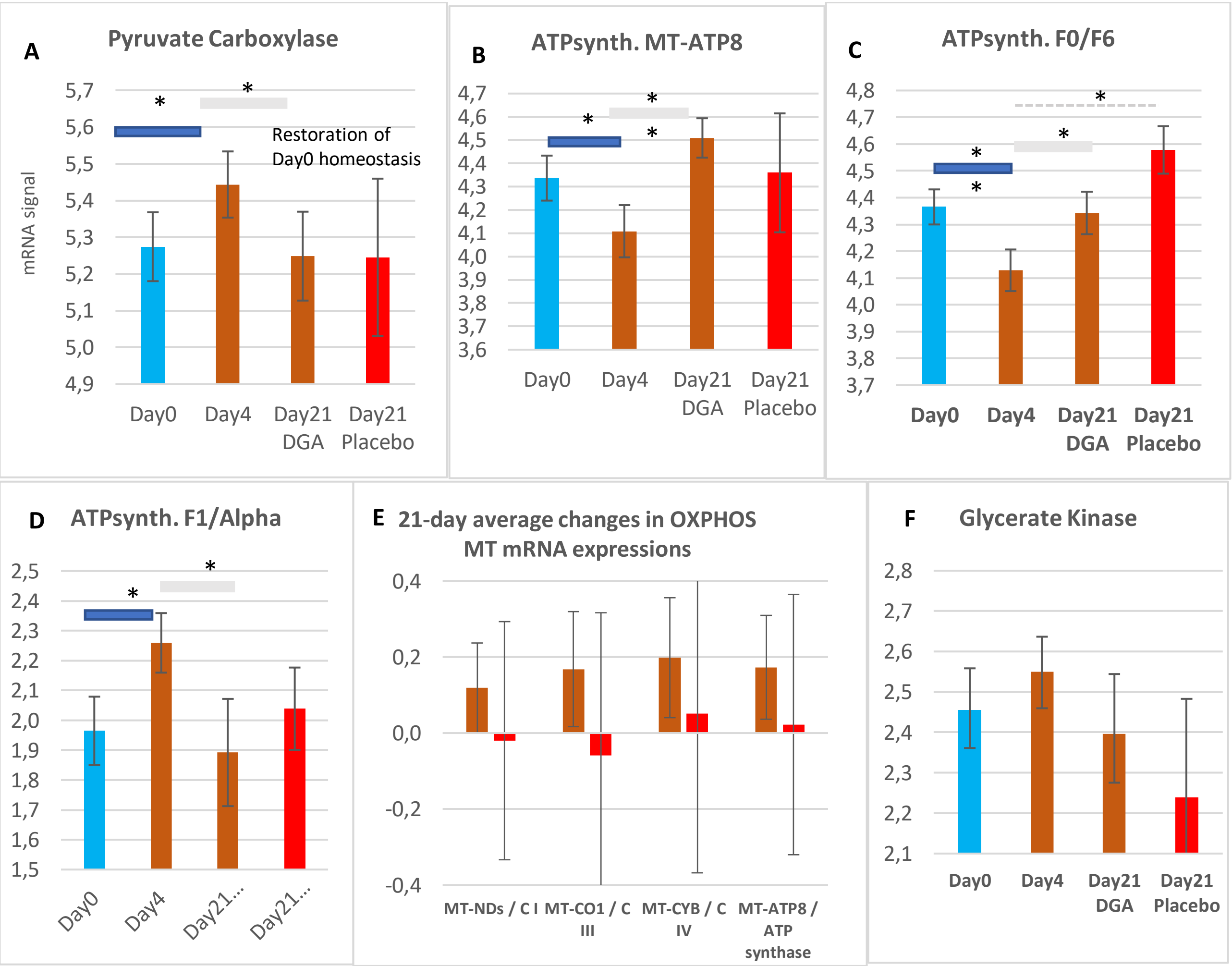

One observation missing in Day4 and Day21 (Fig.D). Grey color (Figs.A-D) indicates that the respective comparison was not in the study plan and dashed grey color (Fig.C) that the number of observations was low. In Fig.E the MT-NDs represents Complex I, MT-CO1 Complex III, MT-CYB Complex IV, and MT-ATP8 represents ATP synthase. (MT-NDs is the average of MT-ND1 - MT-ND6 gene expressions.)

Pyruvate Carboxylase

In mitochondria, pyruvate is metabolized via PDH enzyme into acetylCoA (main pathway) or alternatively via PC enzyme into oxaloacetate (Fig.3). PC route favors anabolic use of pyruvate compared to more energy orientated PDH route. When there is excess supply of acetylCoA (Fig. 3), like at Day4, pyruvate is in increasing amounts directed to the PC route. Temporary excess supply of acetylCoA from e.g., lactate and other energy substrates (Fig.3) was likely the reason why the mRNA expression of PC gene was strongly upregulated under the 4-day DGA regimen (SBFig.**A**).

mRNA changes of OXPHOS complexes

Likely related to the ATP homeostasis requirement, the ATP synthase / Complex V enzymes were rather strongly modulated at Day4 (SBFigs.B-D). ATP synthase MT-ATP8 and F_0F6 mRNA expressions were both modestly but statistically significantly downregulated. At the same time ATP synthase F_1 subunit Alpha was upregulated. In line with MT-ATP8 enzyme, also other mitochondrially coded OXPOS genes mRNA expressions were modestly downregulated at Day4 (data not shown). MT-ATP8 belongs to ATP synthase complex (SBFig.E).

Downregulation of OXPHOS mRNA at Day4 was consistent with the fact that strong energy metabolic activation during the 4-day DGA regimen (Fig.2A) caused an excess supply of NADH molecules for ATP production. Cellular ATP generation must be somehow constrained to match cellular needs. Nuclear-encoded ATP synthase mRNA expressions opposed each other at Day4 (SBFigs.C-D). This difference possibly reflected the nuclear regulation of cellular ATP generation by the ATP synthase complex.

At Day21, the mRNA expressions of all mitochondrially coded OXPHOS -genes were upregulated in the DGA group and mostly downregulated in the placebo (SBFig.E). In contrast, the nuclear-encoded OXPHOS genes, like ATP synthase F_0F6, were modestly downregulated in the DGA group and upregulated in the placebo at Day21 (SBFig.C). The upregulation of mitochondrially encoded OXPHOS genes in the DGA group may be a sign of modest increase in mitochondrial biogenesis in WBCs during the 21-day DGA regimen (SBFig.E).

Glycerate Kinase

GLYCTK mRNA expression was somewhat activated in the WBCs during the 4-day DGA regimen (SBFig.F). GLYCTK enzymes possess several splice variants that are localized into cytosol and mitochondria. Four of the splice variants are located into the mitochondria and 3 into cytosol (Uniprot Knowledgebase). It may be that some mitochondrial GLYCTK splice variant bound to mitochondrial membranes or to the inter membrane space mediates the DGA activation signal. Unfortunately, the signal from the global mRNA sequencing for GLYCTK was rather weak and thus more in-depth analyses on splice variant activations was not possible in present study.
