# Supplementary material for: Randomized Trial: D-Glyceric Acid Activates Mitochondrial Metabolism in 50–60-Year-Old Healthy Humans
Source: Front Aging. 2021 Oct 29;2:752636. doi: 10.3389/fragi.2021.752636 (PMC9261421; doi:10.3389/fragi.2021.752636)
Supplement: Supplementary file 5 [file DataSheet2.doc]

**CONSORT 2010 Flow Diagram**

**Blinded Real Allocation, Day4**

**Analysis**

**Follow-Up**

**Enrollment**

**Blinded Mock-Allocation, Day0**

Assessed for eligibility (n=45)

Excluded (n=15)

♦  Not meeting inclusion criteria (n=14)

♦  Declined to participate (n=1)

♦  Other reasons (n=0)

Analysed (n=16)
♦ Excluded from analysis (n=1) (During the interview at final measurement session Day21, it turned out that one person had initiated a 3-month strict weight loss diet 10 days before the study. This was against “normal living conditions” criteria and causes significant metabolic changes during the study period.)

(*Additionally, one person’s Day0 mRNA sample in the DGA group possessed technical problem and her others samples were not measured at all.)

Lost to follow-up (n=0)

Discontinued intervention (give reasons) (n=0)

Allocated to DGA intervention (n=17)

♦ Received allocated intervention (n= 17)

♦ Did not receive allocated intervention (n=0)

Lost to follow-up (n=0)

Discontinued intervention (n=0)

Allocated to Placebo intervention (n=10)

♦ Received allocated intervention (n=10)

♦ Did not receive allocated intervention (n=0)

Analysed (n=10)
♦ Excluded from analysis: one participant had to negotiate a deal for his company until morning 02:00 before Day4 measurement. Thus, we excluded his Day4 measurements from analyses.

Randomized (n=30)

Allocated to blinded DGA intervention (n=30)

♦ Received allocated intervention (n= 27)

♦ Did not receive allocated intervention (n=3, last minute cancellations at Day0 due to flu etc.)
