# Supplementary material for: Randomized Trial: D-Glyceric Acid Activates Mitochondrial Metabolism in 50–60-Year-Old Healthy Humans
Source: Front Aging. 2021 Oct 29;2:752636. doi: 10.3389/fragi.2021.752636 (PMC9261421; doi:10.3389/fragi.2021.752636)
Supplement: Supplementary file 6 [file Presentation3.pdf]

Presentation 3 / Supplement C

In vitro NAD+/NADH -tests with human hepatocytes

In a separate earlier study with isolated primary human hepatocytes, cellular NAD+/NADH -ratio was measured from three ex vivo caucasian donors (PLATEABLE CRYOPRESERVED FEMALE AND MALE HUMAN HEPATOCYTES, Celsis In Vitro Technologies. Lot numbers: JGM, CDP, and DOO. Lots JGM, CDP, and DOO are from the same persons as used earlier in the increased energy consumption study.

The cause of death for all donors was cardiovascular related but at the same time they possessed normal liver function. Donors were 50-60 year old and their BMIs were in the same range as in the current in vivo study. Important difference was that the DGA or placebo dose with medium was administered 3 hours before the NAD+/NADH measurement, i.e. an acute dose compared to our 12 hours difference from the last dose in current study. Additionally, the hepatocytes were not in a fasting state because they received new nutrition with the change of the medium 3 hours earlier.

NAD+/NADH ratio was measured using MAK037 NAD/NADH Quantification Kit, from Sigma-Aldrich. It estimates at whole cell level separately "NADtotal" and "NADH". "NADtotal" contains both NAD+ and NADH. Thus, NAD+ is calculated by deducting NADH from NADtotal. It should be noticed that the kit can't measure accurately all membrane bound NAD+ or NADH co-enzymes. Thus, the kit is useful in identifying the changes in NAD+/NADH but it does not necessarily tell anything precise about the absolut amounts of NAD+ and NADH.

At the whole cell level, mitochondrial matrix produces most of cellular NADH in most situations. Cytosolic metabolism into pyruvate generates 1 NADH (Fig.3) compared to 4 NADHs in the mitochondrial PDH and TCA reactions. Additionally, FA oxidation produces all its NADH to the mitochondrial matrix (Fig.3).

Reduction of NAD+ into high energy NADH causes measured NAD+/NADH -ratio to decline rapidly. Thus, testing the NAD+/NADH -ratio with or without DGA forms a very efficient test on possible activation of mitochondria against the 0-control / placebo. Tested DGA concentrations were 0 uM ("placebo"), 1.4 uM, and 14 uM (SCFig.A-B). After needed preparations and overnight incuabation, the hepatocytes were cultivated for two days with or without DGA. Last dose with the medium was given according to recommended 24 h + 24 h medium change cycle.

SCFigure A and B. Cellular NAD+/NADH -ratio, 3h after DGA dose with the new medium. Group comparisons are based on parametric t-tests.

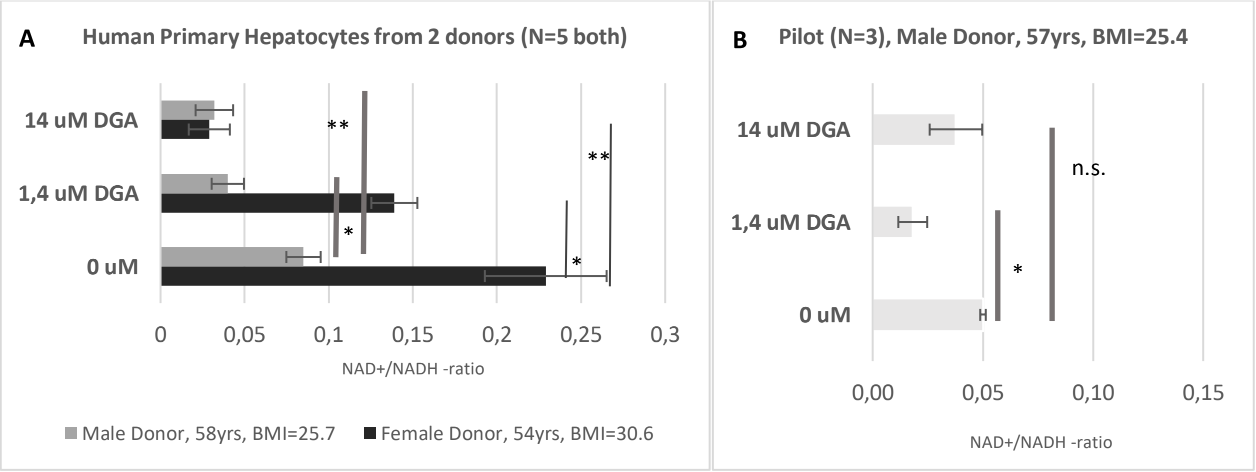

Female donor (5 repeats in each measurement) and male donor (also 5 repeats) received the same DGA doses (0, 1.4 or 14 uM) (SCFig.A) but we modified the amount of received new nutrition with the last

change of the medium down to 1/10th between measurements. Briefly, after experimenting with the female donor (SCFig.A), it was noticed that received excessive nutrition possibly exaggerates results 3 h after. Thus, less nutrition likely explains the downregulation of NAD+/NADH ratio level in the male donor observations (SCFig.A). The pattern was the same in both possibly because also 1/10th of new nutrition 3 h earlier meant fed state for the male donor hepatocytes. Piloting with 1/10th of new nutrition was conducted with hepatocytes of 57 year old human donor (SCFig.B). Same pattern of declining but more modest NAD+/NADH -ratio was received.

Used NAD+/NADH -kit is not optimized for separate measurement and comparisons of NADH but nevertheless we present the NADH results (SCFigs.C-D), which clearly show that there was an upward trend in NADH in the cells treated with 1.4 or 14 uM of DGA compared to the same cells treated with 0 uM (0-control).

SCFigure C and D. Measureable whole cell NADH, 3h after DGA dose with the new medium. Group comparisons are based on parametric t-tests.

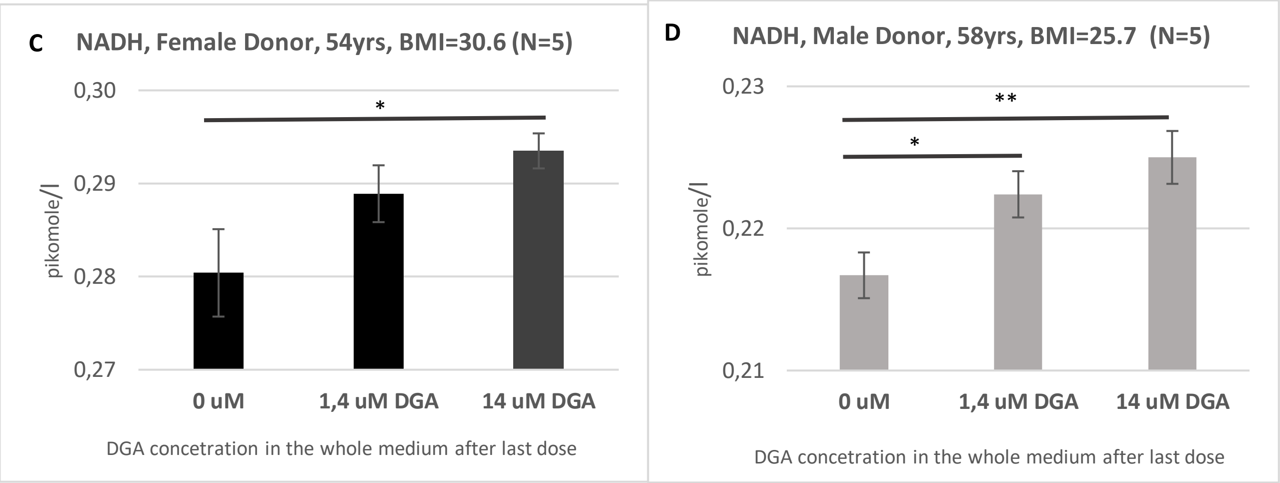

It seems that that after 2 days priming and an acute 3 h treatment with the DGA there was an effect that increased cellular NADH compared to 0-control. Especially NAD+/NADH ratio was strongly and consistently effected. Increased NADH and NADH/NAD+ -ratio in isolated hepatocytes was a clear sign of increased mitochondrial metabolism in the human liver tissues by the DGA regimen. Mitchondria produce more than 80% of NADH and they have to be activated at some point also because mitochondrial OXPHOS is the main site of NADH oxidation.

Based on the results presented in Fig.2A of the article, there was a possibility that the liver was activated only indirectly in vivo (due to increased peripheral tissues energy substrate demand). Now we can conclude that the livers were extremely likely also directly activated in vivo. The interpetation of the results in SCFigs.A-D from mitochondrial activation point of view is rather certain. Otherwise isolated hepatocytes do not possess all the characteristics of hepatocytes in vivo.
