# Supplementary material for: Randomized Trial: D-Glyceric Acid Activates Mitochondrial Metabolism in 50–60-Year-Old Healthy Humans
Source: Front Aging. 2021 Oct 29;2:752636. doi: 10.3389/fragi.2021.752636 (PMC9261421; doi:10.3389/fragi.2021.752636)
Supplement: Supplementary file 7 [file DataSheet1.docx]

# Highlights

- Oral D-glyceric acid (DGA) administration causes both fast and lasting positive metabolic effects in healthy 50-60-year-old humans.
- Overall mitochondrial metabolism was activated by the increase in intracellular DGA concentration
  - both the OXPHOS and NADH using anabolic reactions were upregulated in the whole-body and particularly in immune cells and hepatocytes.
  - Plasma lactate was strongly and statistically extremely significantly reduced.
- Subclinical inflammation measured by 3 independent methods was reduced both in the 4- and 21-days treatments.
- Cellular membrane integrity seemed to be sharply improved. Creatine kinase and aspartate aminotransferase release to plasma was lowered sharply and statistically very significantly already in 4-days.
